# Large-Scale Convergence of Receptor Cell Arrays Onto Afferent Terminal Arbors in the Lorenzinian Electroreceptors of *Polyodon*

**DOI:** 10.3389/fnana.2020.00050

**Published:** 2020-10-19

**Authors:** David F. Russell, Thomas C. Warnock, Wenjuan Zhang, Desmon E. Rogers, Lilia L. Neiman

**Affiliations:** ^1^Department of Biological Sciences, Ohio University, Athens, OH, United States; ^2^Department of Physics and Astronomy, Ohio University, Athens, OH, United States; ^3^Neuroscience Program, Ohio University, Athens, OH, United States; ^4^Honors Tutorial College, Ohio University, Athens, OH, United States

**Keywords:** sensory array, electroreceptors, convergence ratio, afferent terminal branching, myelinated dendrite, ampullary organ, star neural network, terminal glia

## Abstract

Certain sensory receptors contain many transducers, converging onto few afferents. Convergence creates star-topology neural networks, of iterative parallel organization, that may yield special functional properties. We quantitated large-scale convergence in electroreceptors on the rostrum of preadult paddlefish, *Polyodon spathula* (Acipenseriforme vertebrates), and analyzed the afferent terminal branching underlying the convergence. From neurophysiological mapping, a recorded afferent innervated 23.3 ± 9.1 (range 6–45) ampullary organs, and innervated every ampullary organ within the receptive field’s sharp boundary. Ampullary organs each contained ∼665 Lorenzinian receptor cells, from imaging and modeling. We imaged three serial types of afferent branching at electroreceptors, after immunofluorescent labeling for neurite filaments, glial sheaths, or nodal ion channels, or by DiI tracing. (i) Myelinated tree: Each of 3.08 ± 0.51 (2–4) parallel afferents from a cranial nerve (ALLn) entered a receptive field from deeper tissue, then branched into a laminar tree of large myelinated dendrites, parallel to the skin, that branched radially until ∼9 extremities with heminodes, which were candidate sites of spike encoders. (ii) Inline transition: Each myelinated extremity led distally into local unmyelinated arbors originating at inline branching structures covered by terminal (satellite) glia. The unmyelinated transition zones included globular afferent modules, 4–6 microns wide, from which erupted fine fascicles of parallel submicron neurites, a possibly novel type of neuronal branching. The neurite fascicles formed loose bundles projecting ∼105 microns distally to innervate local groups of ∼3 adjacent ampullary organs. (iii) Radial arbors: Receptor cells in an electrosensory neuroepithelium covering the basal pole of each ampullary organ were innervated by bouton endings of radial neurites, unmyelinated and submicron, forming a thin curviplanar lamina distal to the lectin+ basal lamina. The profuse radial neurites diverged from thicker (∼2 micron) basolateral trunks. Overall, an average *Polyodon* electroreceptor formed a star topology array of ∼9 sensor groups. Total convergence ratios were 15,495 ± 6,052 parallel receptor cells per afferent per mean receptive field, assuming 100% innervation. Large-scale convergence likely increases the signal-to-noise ratio (SNR) of stimulus encoding into spiking afferent output, increasing receiver sensitivity. Unmyelinated arbors may also regenerate and repair the afferent innervation of ampullary organs.

**LSID:** urn:lsid:zoobank.org:act:09BCF04C-3C3C-4B6C-9DC9-A2BF43087369

## Introduction

Convergence is a feature of some but not all sensory receptors, whereby the number of sensory transduction elements exceeds the number of primary afferent axons projecting to the central nervous system (CNS). For example, usually 10–20 Merkel cells contact an afferent in the light touch mechanoreceptors of mammalian skin (Lesniak et al., 2014). Certain vestibular afferents likely receive synaptic excitation from 5 to >80 hair cells (Huwe et al., 2015). Visceral afferents may show a high degree of convergence and extensive terminal branching arising from a single axon (Spencer et al., 2014). The numerous receptor cells of an ampulla of Lorenzini electroreceptor (ER) of rays and sharks present an extreme example of convergence onto afferents (Kalmijn, 2000). By contrast, each auditory afferent of mammals contacts only one ribbon synapse of a single hair cell (Fuchs et al., 2003).

Some receptive fields, e.g., in vertebrate retinas, have multiple transducers connected laterally synaptically, e.g., via interneurons, yielding sensory preprocessing such as center-surround properties. Other sensory receptors lack lateral interneurons, and mainly encode the local magnitude of stimuli. In such “scalar” receptors, the functional significance of converging sensors remains unproven. Cost considerations (e.g., potential entry of microbes or parasites into sensory skin pores) suggest that multiple sensors would not be present but for achieving a functional advantage. Data from diverse sensory receptors support a positive correlation between sensitivity and the count of sensors (Corwin, 1983; Meisami, 1989; Sanchez and Zakon, 1990; Peters et al., 1997), although the scale of this positive correlation varies widely. Apart from studies on retinas (Baden et al., 2018), neural mechanisms for the positive correlation remain unclear or hypothetical.

Electronic sensor arrays with repeated spatially distributed transducers have advantages for signal reception (van Trees, 2002), and are used in diverse fields, e.g., audio recording (Benesty et al., 2008). Examples of neural sensory arrays include retinas, the lateral line of aquatic vertebrates (e.g., Coombs and Conley, 1997), and sensillae of insects (e.g., Cogley, 1999). Each electroreceptor of *Polyodon*, which we studied, comprises a sensory array.

**FIGURE 1:**
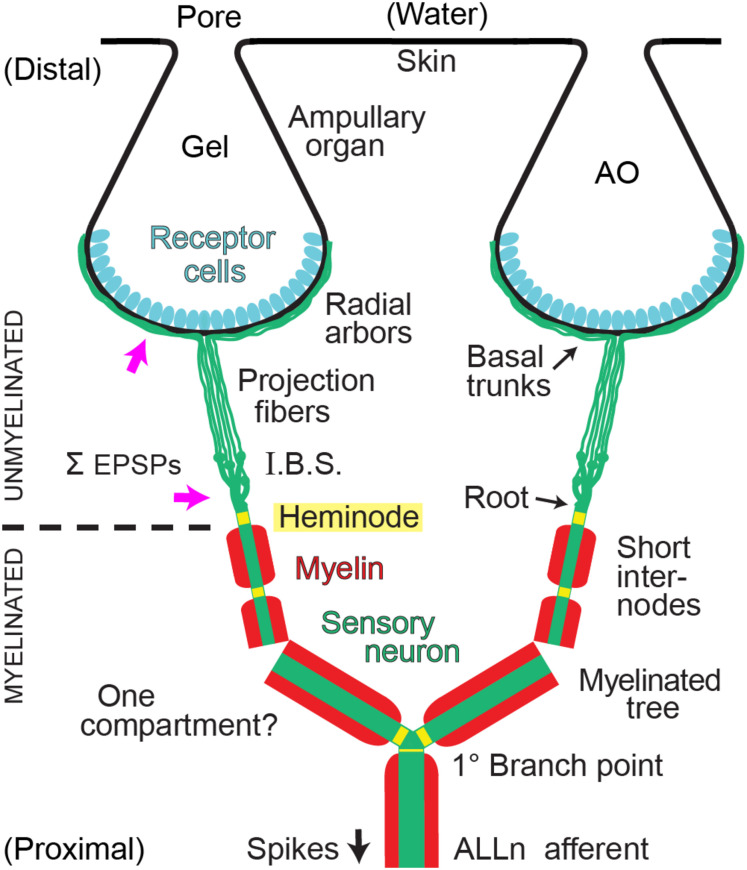
Summary of afferent branching at a *Polyodon* electroreceptor. Distal ampullary organs (AO) were innervated by local unmyelinated arbors from all afferents. Central myelinated (red) arbors, located deeper, received input from all ampullary organs in a receptive field. ALLn, Anterior lateral line cranial nerve. I.B.S., Inline branching structure, the origin of an unmyelinated arbor, converged onto its root. Pink arrows indicate sites where summation (Σ) of excitatory postsynaptic potentials (EPSPs) from receptor cells likely occurs. One compartment?: The wide dendrites of the central myelinated tree of each ALLn afferent likely form a strongly coupled electrotonic compartment.

Myelinated primary afferents of vertebrates undergo structural transformations as they approach and innervate peripheral sensory organs, and always end distally as unmyelinated neurites. For example, the myelinated afferents of mammalian light touch mechanoreceptors branch into multiple myelinated dendrites ending at heminodes, then short (∼10 μ) branched unmyelinated neurites lead to sensory terminals at Merkel cells (Lesniak et al., 2014; Marshall et al., 2016). Myelinated bouton afferents to vestibular neuroepithelia branch, distal of a basal lamina, into unmyelinated neurite arbors of 50–100 μ extent (Fernández et al., 1995). We imaged unmyelinated terminal neurites that were long and profusely branched (summarized in Figure 1).

**FIGURE 2:**
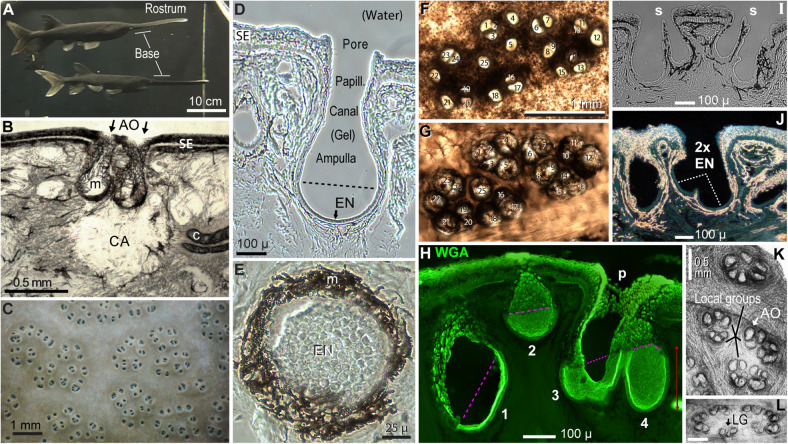
*Polyodon* electroreceptors. **(A)** The rostrum and its base are labeled on two paddlefish in the colony aquarium. **(B)** This cross section of rostrum skin illustrates a clear area (CA) under ampullary organs (AO, arrows), at gaps in subdermal cartilage plates (c). m, Melanin pigment in fibrous AO walls. SE, Striated ectoderm skin. **(C)** Electroreceptor skin pores on the rostrum base of a live paddlefish in water. **(D)** Cross section of one ampullary organ. EN, Electrosensory neuroepithelium, below the dashed line. Papill., Lozenge-shaped papilla cells lined the canal and pore. **(E)** Planar patch of electrosensory neuroepithelium (EN) in a section that grazed the wall of an ampullary organ. **(B,D,E)** Unstained phase contrast single images of tissue sections. **(F,G)** Correspondence 1:1 of 25 skin pores in a cluster **(F)** to 25 ampullary organs of the same cluster **(G)**, in a vibratome section cut parallel to skin at ∼400-μ depth. Panel **(F)** was left–right reversed for direct visual comparison to panel **(G)**, with corresponding numbering. Panel **(F)** was imaged from above by transillumination after stripping off a superficial pigmented layer. Panel **(G)** was imaged from below to reveal AOs as basal outpocketings delineated by melanin-pigmented fibrous connective tissue. **(H–J)** Variants of AOs. **(H)** AOs of different sizes (#1–4) were stained by wheat germ agglutinin (WGA) lectin in this stack projection from a 50-μ section. The superficial borders of electrosensory neuroepithelia were marked by pink dashed lines. p, Papilla cells lined the AO neck and pore. **(I)** Thin septa (s) separated pairs of AOs sharing common skin pores; brightfield image. **(J)** This large skin pore led to two electrosensory neuroepithelia (2x EN). Darkfield illumination revealed melanin pigment as gold-colored. **(K,L)** AOs formed subgroups **(K)** or a perimeter **(L)**, in *en face* transilluminated images of the deep faces of thick vibratome sections parallel to skin, bleached in H_2_O_2_. Calibration in **(L)** was 0.5 mm. LG, Local group of three ampullary organs.

We analyzed convergence in the ampullary electroreceptors on the rostrum of paddlefish (*Polyodon spathula*). Their rostrum is a horizontally flattened electrosensory appendage anterior of the head (Figure 2A), covered with many ERs. Specialized ERs cover other anterior body surfaces also. Paddlefish use passive electrosense to localize zooplankton prey (Russell et al., 1999; Wilkens et al., 2001). The ancestral “Lorenzinian” ERs of *Polyodon* (and related sturgeons) are homologous with the ampulla of Lorenzini ERs of sharks and rays (Jørgensen, 1989; Baker et al., 2013).

*Polyodon* ERs are two-stage sensory receptors, like others of the lateral line and inner ear. Receptor cells are contained in ampullary organs (AOs), embedded in striated ectoderm skin. Each ER on the rostrum includes multiple AOs, grouped in a cluster 0.5–2 mm across, and a small group of afferents innervating them (Neiman and Russell, 2004; Chagnaud et al., 2008). Each ER is innervated by a branch of the ipsilateral anterior lateral line (ALLn) cranial sensory nerve (Allis, 1920; Norris, 1925). Electroreceptor afferents originate from sensory neuron somata in discrete ALLn ganglia near the brain, and project centrally to the medullary dorsal octavolateralis nuclei (Hofmann et al., 2002).

Each AO (where electrosensory transduction occurs) has a skin pore leading into a gel-filled canal and expanded deeper ampulla (Figures 2B–D). Its basal interior surface is covered by a one-cell-thick electrosensory neuroepithelium (*EN*, Figures 2D,E) (Jørgensen et al., 1972; Modrell et al., 2017) containing receptor cells specialized for sensing weak external voltage signals (Bellono et al., 2017), along with support cells. The basal poles of receptor cells form multiple excitatory ribbon synapses, presynaptic to bouton-like postsynaptic contacts of afferents, described by EM (Jørgensen et al., 1972).

We quantitated the number of AOs in ER receptive fields on the rostrum by functional mapping using microstimulation of skin pores. The receptive field of a recorded single-unit ALLn afferent was defined as the set of AOs modulating the afferent’s background firing. Neighboring ERs on the rostrum often cannot be reliably distinguished by surface inspection of skin pores.

Our study of convergence in *Polyodon* ERs includedmorphological analyses of the number of receptor cells per AO, the number of ALLn afferents, and structural motifs in the peripheral terminal branching of ALLn afferents. Fluorescence image stacks were collected after immunolabeling or DiI tracing. We imaged three distinct types of afferent terminal arborizations (Figure 1), that mediate different components of sensory convergence. The arborizations included (i) radial unmyelinated arbors on individual AOs, (ii) inline branching at unmyelinated transition zones that innervate local groups of a few AOs, and (iii) myelinated central radial arbors that collect electrosensory input from an entire receptive field. Their serial ordering achieves high divergence by which individual afferents innervate ∼10^4^ receptor cells in a receptive field.

Our data suggest functional implications. Large-scale convergence may increase the signal-to-noise ratio (SNR) of stimulus encoding into spiking afferent output, increasing the receiver sensitivity of *Polyodon* ERs. The unmyelinated terminal arbors of afferents may also comprise a regeneration system for repair of ER innervation.

## Results

Counts or measurements are reported as mean ± SD for *v* values from *n* paddlefish. The numbers of tissue applications are noted as *t* for different molecular labels in Table 1. In most figures, the distal/apical/superficial direction toward the skin is shown as “up.” The figures present multiple examples to illustrate variation.

**TABLE 1:** Primary antibodies (Ab) used.

Abbreviation	Antigen, Name	Host	Manufacturer	Cat. #	RRID	*t*	*n*
aTub	Acetylated α-tubulin (clone 6-11B-1)	M	Santa Cruz	sc23950	AB_628409	>10	6
—	*N*-cadherin (C-terminus)	R	GeneTex	GTX127345	—	4	2
CALB1	Calbindin (partial sequence)	R	GenScript	A01268	AB_1582512	>10	4
K_*V*_1.1	Potassium ion channel 1.1	R	Alomone	APC-009	AB_2040144	>10	6
MBP	Myelin basic protein	R	GenScript	A01407	AB_1720890	>10	6
Nefh	Neurofilament 200 kDa	C	Aves	NFH	AB_2313552	>10	6
Na_*V*_	Sodium ion channels (pan partial sequence)	M	Sigma	S8809	AB_477552	>10	6
NMDAR1	*N*-Methyl-D-aspartate receptor 1	R	GenScript	A01587	AB_1968883	3	2
PVALBα	Parvalbumin-α	R, C	GenScript, Encor	A01439, CPCA-Pvalb	AB_1720924, AB_2572371	>10	4
P0	Protein zero	R	Neuromics	CH23009	AB_1619444	>10	2
—	S100β	R	GeneTex	GTX129573	—	2	1
ZO1	Tight junction protein 1	R	GeneTex	GTX108613	AB_1952257	>10	5

*C, chicken yolk polyclonal Ab. M, mouse monoclonal Ab. R, Rabbit affinity purified polyclonal Ab. The numbers of tissue applications are noted as t, to tissue from n paddlefish. The anti–Na_*V*_ monoclonal Ab was clone K58/35.*

Our fluorescence imaging of afferent terminals was facilitated by the transparency and low background of a “clear area” (*CA*, Figure 2B) under each electroreceptor on the rostrum of *Polyodon*. Such dome-shaped pockets contained unusual translucent loose connective tissue, with dispersed small fibroblasts. This gel-like soft tissue occupied gaps or channels in subdermal cartilage plates (*c*, Figure 2B), or spaces between stellate bones (Grande and Bemis, 1991). Clear areas extended to depths of 1.13 ± 0.19 mm below the skin surface (from nine ERs, four fish), in the same strata where we labeled and imaged afferent terminal arbors. Small nerves branching to ERs were visible in such clear tissue without staining.

### Correspondence of Skin Openings to Ampullary Organs

To support functional mapping, we assessed the correspondence of skin pore openings to ampullary organs and electrosensory neuroepithelia.

Ampullary organs on the rostrum varied in size. The AOs in Figure 2B were representative of usual “deep” AOs extending to 350–500 μ below the skin surface. The AO in Figure 2D, with a distinct canal, was unusually deep (580 μ). Another class of AOs was smaller (e.g., #2 in Figure 2H), extending to 258 ± 43 μ interior depth (*v* = 26), measured to the apical surface of their EN.

A skin pore could lead to a single AO (Figure 2D). However, many other skin “pores” had septate subdivisions into 2–4 smaller openings (Camacho et al., 2007), e.g., #8 + #9 and others in Figure 2F. Cross sections verified that the septate subdivisions of pores led to separate AOs (Figure 2I), although their bulbous ampullas touched. Hence, in many receptive fields, the “skin openings” corresponded 1:1 to ampullary organs (Figures 2F vs. 2G), each with one EN.

**FIGURE 3:**
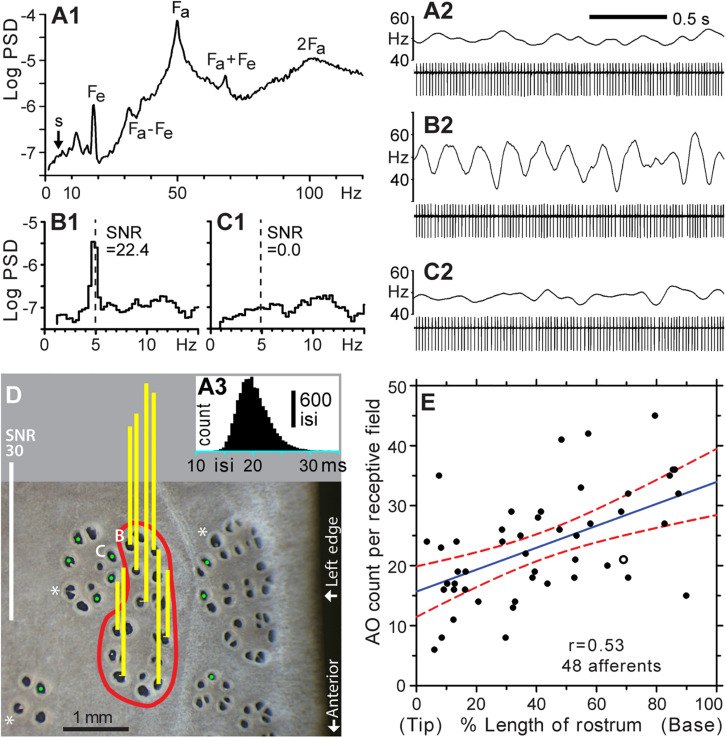
Functional mapping of receptive fields by microstimulation of individual ER pores. **(A1–A3)** Background firing of an ALLn sensory neuron without external stimulation, at mean 49.7 Hz. **(A1)** Power spectral density (PSD, logarithmic scale, ∼0.31-Hz binwidth), was calculated from 14,924 spikes, 5-min data. **(A2)** Raw recording, 2 s. **(A3)** Histogram of all interspike intervals (isi) in 5-min data; 0.5-ms bins. The single-unit afferent was recorded extracellularly via a microelectrode in an ALLn ganglion, and digitized at 20 kHz. The PSD was calculated from a derived 20-kHz sampled voltage channel of zero values with a single 1-V sample at each detected spike time. Labels mark the fundamental frequency of an afferent oscillator (Fa) and epithelial oscillations (Fe), and combinations or multiples of these (Fa + Fe, Fa – Fe, 2Fa). **(A2,B2,C2)** Spike amplitudes in filtered (100–1000 Hz) recordings were approximately 510, 460, or 640 μV, respectively. Firing rates (upper trace in each panel) were smoothed in Spike v7 software by summing a unit-area Gaussian waveform, of 50 ms-SD and 200-ms total width, at each spike time. **(B1,2)** Modulation of afferent firing from 10 cycles of 5 Hz, ±2.5 nA peak–peak, sine wave stimulation of the pore labeled **(B)** in panel **(D)**. **(B1)** PSD from 2,870 spikes, 1-min recording. The signal:noise ratio (SNR = 22.4) at 5 Hz was calculated as the ratio of summed net (total minus noise) response power in three signal bins centered on 5 Hz, to the summed extrapolated power of baseline noise in these three bins, averaged from below and above the response band. **(B2)** Raw recording, 2 s, 10 stimulus cycles. **(C1,2)** Nil response (SNR = 0.0) of a pore in an adjacent cluster, labeled **(C)** in **(D)**, for 5 Hz ± 2.5 nA sine wave stimulation (dashed line). **(C1)** PSD from 2,828 spikes, 1-min recording. **(C2)** Raw recording, 2 s, 10 stimulus cycles. **(D)** This photo of skin pores on the rostrum surface shows the receptive field (red boundary) of the afferent unit for panels **(A–C)**. Sensitivity for 5 Hz ± 2.5 nA sine wave stimulation was measured at every skin opening within the red boundary; yellow bars show response SNR at selected pores. Pores in the receptive field were defined by SNR > 9, measured as in **(B)**. Pores outside the receptive field yielded nil response (SNR ∼ 0, marked by green dots, 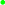).*, Large pores with partial septa. **(E)** Counts of ampullary organs (AO) in the receptive fields of 48 different afferents were mapped on the left-side dorsal surface of the rostrum of six paddlefish of ∼1-year age. The blue solid line shows the least squares regression (correlation coefficient *r* = 0.53), and red dashed curves shows its 95% confidence intervals, from SigmaPlot software. The *x* axis shows locations of the mapped receptive fields along the rostrum length, expressed as percentage of distance from the rostrum tip (0%) to the dorsal nares (100%). The open circle corresponds to the receptive field in **(D)**.

An exception were some extra-large skin pores (>100 μ), common on the rostrum’s base. Some had an incomplete septum (*, Figure 3D), while others showed only a ridge between what appeared like EN pairs (Figure 2J). Hence some large pores led to two ENs. This suggested that AOs may undergo a maturation process whereby adjacent AOs tend to fuse as paddlefish age.

### Functional Mapping of Receptive Fields

Accepting 1:1 correspondence of skin openings to ampullary organs as typical, we used functional mapping to count the mean number of AOs in the receptive fields of ERs on the rostrum, needed to quantify convergence. Prior reports (Wilkens et al., 1997; Neiman and Russell, 2004; Chagnaud et al., 2008) showed (i) that afferents to ERs on the rostrum were indeed from sensory neurons of the ALLn cranial nerves (Allis, 1920; Norris, 1925), (ii) that the receptive fields of ALLn single units corresponded to natural clusters, ∼0.5 to ∼2 mm across, of sensitive skin pores on the rostrum, and (iii) that an individual ALLn afferent innervated only one local sensitive area on the rostrum, never multiple widely separated areas. However, the mean number of AOs in the receptive field of an ER on the rostrum has remained unknown. Here, the receptive field of an ER afferent was defined as the set of AOs which, when microstimulated individually and weakly, elicited modulation of the afferent’s background firing.

Without stimulation, a single-unit ALLn afferent fired continuously and quasiperiodically at ∼50 Hz, attributed to ongoing synaptic excitation by receptor cells. Power spectra of such background firing (Figures 3A1,A2) showed peaks at intrinsic oscillator frequencies (*Fa*, *Fe*) and at their side bands and multiples (Neiman and Russell, 2004). This continuous background firing of a recorded single afferent unit was modulated by weak external sine wave stimulation from a local electrode, yielding a line-like power spectrum peak at the 5-Hz stimulus frequency (Figures 3B1,B2). The ∼5-Hz frequency band presented a rising baseline (*s*, Figure 3A).

We confirmed that sensitive AOs (i.e., which modulated the background firing of a recorded afferent unit) were clustered together in rosettes. An afferent unit could be held long enough (∼2 h) to measure the SNR at every skin opening in a sensitive cluster, along with spot-checking of insensitive pores in surrounding morphological clusters. Figure 3D shows representative results of mapping one receptive field on the rostrum (*red boundary*), identified as an elongated cluster of 21 skin openings. All of its pores were tested, and each drove firing of the recorded ALLn afferent. Vertical bars over selected pores show responses as the SNR of afferent spiking power at 5 Hz, responding to in-pore sinusoidal stimulus currents of ±2.5 nA peak–peak. Positive responses (*yellow bars*) had SNR = 23.9 ± 10.2 (range 9.2–41.4), compared to SNR ∼ 0 at pores outside (*green dots*, 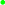). This robust difference allowed binary identification of AOs (skin openings) as innervated or not by a recorded single-unit ALLn sensory neuron.

Counts obtained by such functional mapping yielded 23.3 ± 9.1 skin openings of AOs (range 6–45) per receptive field (Figure 3E), for the different receptive fields of 48 (total) recorded single-unit ALLn afferents innervating the left-side dorsal surface of the rostrum, from *n* = 6 fish. These raw counts were not corrected for large pores having more than one EN. That is, each skin opening was counted as one AO, whether of a single pore, a septate subdivision of a pore, or a large pore. The mapped receptive fields were located throughout the length of the rostrum. The largest count (45) was found posteriorly on the rostrum base, and the smallest count (6) was found near the anterior tip, but counts showed only a weak statistical trend to increase along the rostrum length (*blue regression line*, correlation coefficient *r* = 0.53).

Although the test stimulus voltage applied to different AOs likely varied due to uncontrolled shunting at pores, the functional approach used here sufficed for defining the set of AOs included in an afferent’s receptive field.

#### New Data on Receptive Field Organization

We found that the pores (AOs) of different ERs were not intermingled. A recorded afferent’s sensitive pores were always adjacent to other sensitive pores, in rosette-like clusters, and did not occur as separate outlying individual pores. Thus, there were sharp borders between adjacent ERs, marked by abrupt transition to nil afferent response at pores outside of a sensitive cluster (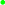, Figure 3D). For example, the pair of adjacent pores marked as “B” and “C” in Figure 3D corresponded to the data of Figures 3B1,B2 or 3C1,C2 respectively; pore “B” was within the receptive field, whereas pore “C” was outside. They showed no crosstalk at ±2.5 nA peak–peak (maximal) stimulation, despite being close together, verifying (as a control) that a pore and its AO and EN were stimulated individually.

On the rostrum near midlength, a functionally mapped receptive field often formed a single natural morphological cluster that was discrete and well-separated from other clusters. By contrast, on the rostrum’s base, recorded afferents responded to stimulation of AOs in two to four adjacent morphological rosettes. Clusters on the base were packed close together (Figure 2C), so different ERs could not be distinguished by surface inspection of skin pores.

We found that usually every skin opening tested as sensitive (SNR > 9) within the borders of a functionally mapped receptive field. A few skin pores near the center of a sensitive cluster could show lower sensitivity, but out of ∼1,000 tested, only one skin pore proved insensitive within an otherwise sensitive rosette. Hence, as a rule, a given afferent innervated every AO within the well-defined border of its receptive field. This was important for our study of afferent innervation (below). By extension, the parallel afferents to an ER likely innervate identical sets of AOs.

### Receptor Cell Quantity Estimates

A key metric for our analysis of convergence was the number of receptor cells in one ampullary organ, in the electrosensory neuroepithelium covering the inside of each AO’s basal pole. For this, we measured the EN area per receptor cell, and modeled the apical surface area of ENs from their dimensions in cross and parallel sections.

#### Apical EN Area per Receptor Cell

**FIGURE 4:**
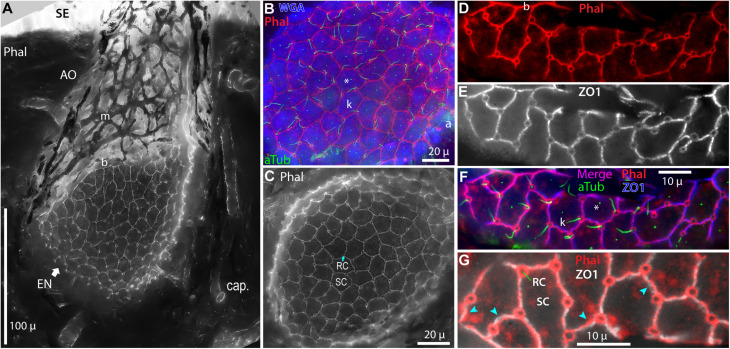
Intercellular junctions on the apical surfaces of electrosensory neuroepithelia, between receptor cells (RC) and support cells (SC), were labeled by phalloidin (Phal) or anti–ZO1, and imaged as projections from stacks. **(A)** This tangential cross section of an ampullary organ (AO) exposed a large expanse of the apical surface of the electrosensory neuroepithelium (EN), extending to its superficial border (b) with the canal wall. Capillaries (cap.) surrounded the AO. m, Melanin-pigmented mesh of fibers in the AO wall. **(B)** Phalloidin (Phal) revealed cell borders on the apical surface of this triple-labeled pseudocolored EN. Wheat germ agglutinin (WGA) labeled the apical surfaces of support cells (but not receptor cells). Anti–acetylated α-tubulin (aTub) labeled kinocilia (k) on receptor cells, and also labeled afferent (a) branches, and short cilia (*) on support cells. **(B,C)** These large expanses of EN did not include borders with canal wall, and so were basal in their AOs. **(D–G)** Colocalization of labeling by phalloidin and anti–ZO1. **(F,G)** Images of different labels were aligned by applying offsets of fluorescence channels based on calibrations from TetraSpeck fluorescent beads. **(F)** Kinocilia (k) on receptor cells were labeled by anti–aTub to identify the apical membranes of receptor cells; anti–aTub also labeled short cilia (*) on support cells. **(G)** Co-labeling of intercellular junctions by phalloidin and anti–ZO1 revealed general colocalization, but with local mismatches or small offsets (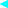). **(A,C)** 40x 0.75 NA water lens. **(B)** 60x 1.4 NA oil lens. **(D–G)** 100x 1.25 NA oil lens, 0.4 μ stack step size.

We labeled and imaged *en face* the electrosensory neuroepithelia of ampullary organs in widefield stacks of frozen sections that cut some AOs in a glancing fashion, exposing a near-planar portion of an EN (Figures 2E, 4A–C), on the inside of an expanse of AO wall. Fluor-conjugated phalloidin was an effective label for the apical-surface junctions between support cells and receptor cells (Figure 4) (Modrell et al., 2017). Phalloidin+ junctions formed lines, circles, or polygons. Phalloidin+ junctions were also labeled by anti–ZO1 (Table 1 and Figures 4D–G), a criterion component of tight junctions, although local offsets and small differences were discernible (Figure 4G). Hence phalloidin labeling revealed the zona occludens tight junctions around support and receptor cells apically, known to be crucial for electrosensitivity (Obara and Bennett, 1972). Phalloidin and anti–ZO1 also labeled tight junctions between all canal wall cells.

Both phalloidin and anti–ZO1 labeled a small circle of zona occludens tight junctions around the apical pole of each receptor cell (*RC*, Figure 4). A long kinocilium (*k*, Figures 4B,F) labeled by anti–acetylated α-tubulin (aTub, Table 1) was aligned within each small circle, confirming its identity as the apical membrane of a receptor cell (Jørgensen et al., 1972). We measured the mean area of the apical membranes of receptor cells (Table 2, #1) as equivalent to ∼1.5-μ diameter (for a circle of equal area), similar to values from a marine ray (Wueringer et al., 2009). The apical membranes of receptor cells comprised only 3.3% of the total apical EN surface area.

**TABLE 2:** Measured values of *Polyodon* ERs.

	Quantity	Mean	*SD*	Range	Unit	*v* (from)	Notes
1	Area of RC apical membrane	1.77	0.13	1.67–2.03	μ^2^	538 RCs (19 EN)	Traced along middle of phalloidin+ border
2	Area of SC apical membrane	103.3	11.3	91.4–119.2	μ^2^	350 SCs (18 EN)	Traced along phalloidin+ border
3	Inverse apical density of RCs	54.98	3.65	50.77–63.32	μ^2^ /RC	18 EN (4 fish)	From central image area containing 28.3 ± 4.2 RCs
4	Inverse areal density of RCs	59.17	5.61	52.94–68.44	μ^2^ /RC	10 EN (4 fish)	From parallel optical sections within EN; 30–164 RCs per EN
5	Parent neurite widths in EN	0.64	0.18	0.39–1.37	μ	36 parents	All binary branch points
6	Progeny neurite widths in EN	0.47	0.13	0.26–1.06	μ	72 progeny	All binary branch points
7	Projection bundle minimum width	20	6.7	14.3–37.2	μ	14 bundles (10 ERs)	Measured near midlength in 50-μ frozen sections
8	Projection fiber widths	0.84	0.30	0.42–2.26	μ	162 (20 images)	Measured in 4x resampled images
9	Unitary projection neurite width	0.37	0.06	0.26–0.44	μ	9 neurites (4 ERs)	60x 1.4 NA or 100x 1.25 NA objective lens
10	Transition zone inline lengths	53	11	34–72	μ	63 zones (21 ERs)	Between start of projection fibers and MBP+ internodes
11	IBS ovoid major axis width	6.1	2.1	—	μ	23 ovoids (7 ERs)	For ellipses fitted in ImageJ to traced outlines of ovoids
12	IBS ovoid minor axis width	3.8	1.0	—	μ	23 ovoids (7 ERs)	For ellipses fitted in ImageJ to traced outlines of ovoids
13	Path length of IBS root segments	10.3	6.1	up to 20.4	μ	25 roots (12 ERs)	Between 1st branch point of IBS and start of MBP+ labeling
14	Maximum width of IBS root segments	4.3	1.3	2.4–7.8	μ	25 roots (12 ERs)	Near the start of myelin on terminal internodes
15	Minimum width of IBS root segments	2.2	1.1	0.6–4.9	μ	25 roots (12 ERs)	Near midlength
16	Ratio of minimum IBS root width	0.52	0.21	0.15–0.93		25 roots (12 ERs)	Normalized to maximum root width near myelin
17	Ratio of widths, progeny/parent	0.66	0.13	0.44–0.97		89 (7 ERs, 4 fish)	33 Branch points; each had two to six progeny myelinated dendrites
18	Ratio of summed progeny areas to parent area	1.23	0.35	0.48–2.21		33 (7 ERs, 4 fish)	Transverse circular areas were calculated from widths

*AO, ampullary organ; EN, electrosensory neuroepithelium; RC, receptor cell; SC, support cell. Lines 3, 4: A measured area was defined by drawing a perimeter in Image J between centers of receptor cells; those on the perimeter were counted as 0.45 RC to compensate for edge effects. Line 9: The width of each unitary peak was measured near its base, at 15% of peak height above extrapolated baseline. Lines 17, 18: Notes apply to both; the widths of sensory neuron processes were measured at 50–100 μ from a branch point’s center, to be outside of the narrowed branch point processes.*

Most (96.7%) of the apical EN surface area was occupied by support cells (*SC*, Figure 4 and Table 2, #2), often of pentagonal or hexagonal shapes. Short cilia on support cells were labeled by anti–aTub (*, Figures 4B,F). The apical membranes of ∼6 receptor cells were located around the perimeter of each support cell, usually at its polygonal vertices.

From *en face* imaging of phalloidin-labeled ENs, the apical area of EN per receptor cell (i.e., their inverse areal density) was 54.98 ± 3.65 μ^2^ (Table 2, #3). A similar estimate came from an alternate method, by counting the rounded profiles of receptor cells in optical sections aligned parallel within the interior of ENs stained by lectins (Table 2, #4).

#### Apical Surface Area of Electrosensory Neuroepithelia

Along with the inverse areal density of receptor cells, the average total apical EN surface area was needed to estimate the number of receptor cells in one average EN. To quantify their shapes, the width and depth of ENs in 105 AOs were measured in cross sections, to the apical surfaces of visible receptor cells (Table 3A). An attempt was made to match a representative AO composition of receptive fields, by including in the sample 23% as small-type AOs, and 10% as large-pore AOs having two ENs (hence *v* = 116 ENs overall). Only cross sections of AOs with open canals and visible skin pores were measured, hence near their middle with near-maximal dimensions.

**TABLE 3:** Estimates of receptor cell quantity.

(A)	Mean	SD	Range	*v*	#	(B)	EN area	# RCs	# RCs	# RCs
Quantity					Sec.	Model	(μ^2^)	per EN	(SD)	(Range)
EN semi-width (μ)	80.1	21.4	28–150	116	73	Hemispheroid	36,736	668	−229 / +266	92 – 2,447
EN depth (μ)	69.3	29.1	15–183	116	73	Hemitriaxial ellipsoid	36,579	665	−226 / +263	—
Major/minor AO axis ratio	1.35	0.19	1.02 –1.94	229	12					

***(A)** The depths and maximum widths of 116 electrosensory neuroepithelia (EN) were measured from brightfield or lectin-stained frozen cross sections (Sec.) of skin, by drawing a line between the superficial apical borders of visible receptor cells as the width, then measuring depth orthogonally from the line to each EN’s apical surface, bisecting the enclosed area (as Figure 2D). The 105 ampullary organs (AO) included 11 with large pores and pairs of EN, yielding a total of 116 EN. Data were from five paddlefish. #, v, Number of values. The ratio of the major and minor axis widths of AOs were measured by tracing their outlines, then fitting ellipses in ImageJ, on resampled images of thick vibratome sections parallel to skin (as Figures 2K,L). **(B)** The number of receptor cells (RCs) per EN was estimated from two models of EN apical surface area. Using online calculators, the surface area for an oblate hemispheroid model was calculated from the mean measured semi-width and depth of 116 ENs (A). The surface area for a triaxial hemispheroid model was calculated using Knud Thomsen’s approximate formula with an exponent of 1.6075, from the mean measured depth and the estimated major and minor axis semi-widths obtained by apportioning algebraically the mean width, assumed to be an average of ellipse widths, based on a mean AO ellipticity ratio of 1.3457 (A). Surface areas from the two models were divided by 54.984 μ^2^/RC (Table 2, #3) to estimate the number of RCs per EN. SDs of the number of RCs per EN were estimated, for the two models, from surface areas based on the mean measured semi-width (A) plus or minus 1 SD. Range values for the number of RCs per EN for the hemispheroid model came from calculating surface areas individually for 116 EN, as oblate or prolate hemispheroids, based on their measured individual semi-widths and depths. Averaging the individual surface areas yielded an estimate of 708 RCs per EN, ∼6% more than in (B) (based on mean width), due to nonlinearity of surface area. Range values were not calculated for individual hemiellipsoid models because the estimation of ellipse axes assumed the mean width of ENs.*

**FIGURE 5:**
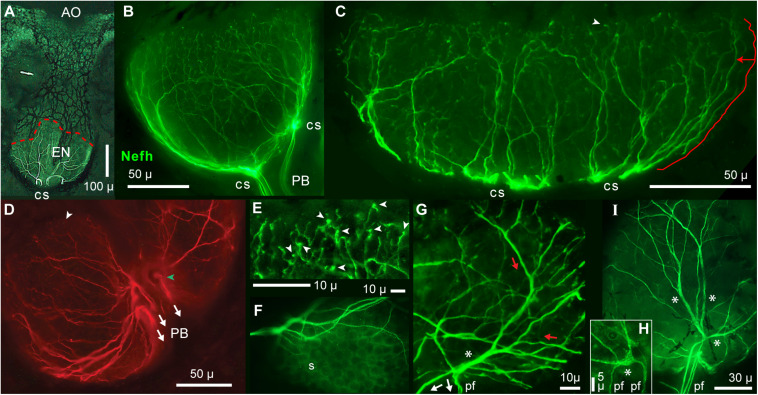
Radial unmyelinated afferent arbors on electrosensory neuroepithelia (EN). All panels show immunolabeling of afferents by anti–neurofilament-H (Nefh). **(A)** Overview of a whole ampullary organ (green, brightfield) superimposed with the ramifying afferent innervation labeled by anti–Nefh (white), and basolateral contact sites (cs) of projection bundles. The dashed red line follows the EN’s superficial border. **(B,C)** Lateral views of afferent trees (green) on the electrosensory neuroepithelia of a small or large AO. **(C)** The length of the marked path along a dendrite (offset red line), to the EN’s superficial border, was measured segment-wise in ImageJ; see DISCUSSION text. **(D)** This basal view of Nefh+ radial afferent trees on an EN shows connections to three fascicles of projection fibers (arrows) at focal contact sites on the basal AO face, near its center. 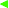, Surface marking. **(E)** This enlarged image of bouton-like endings (arrowheads), presumably at postsynaptic sites on receptor cells, was from the superficial (upper) edge of the arbor in **(B)**. Other bouton endings are marked in **(C,D)** (arrowheads). **(F)** Circular “sockets” (s) surrounded receptor cells. **(G)** Enlarged view of afferent “branching” on an EN. Red and white arrows show presumed directions of EPSP current flows. A basolateral segment (*) of enlarged diameter was the centric root of an extensive unmyelinated afferent tree (red arrows) distally. This basolateral segment connected to the ends of three projection neurites (pf), identified from the full-size image. **(H)** This local Nefh+ swelling (*), ∼3 μ across, Nefh+ throughout, was in-between three dendrites (upper) and two basal projection fibers (pf) identified from the full-size image. **(I)** Long enlarged Nefh+ segments (*) ramified distally (upward) from basal contact sites of four projection fibers (pf). The elongated basolateral segments were wider (1.4–2.4 μ) than the projection fibers leading to them (0.67–1.1-μ widths). **(B–E,G–I)** 40x 0.7 NA lens. **(F)** 60x 1.4 NA lens.

In lateral views (cross sections), an EN typically expanded superficially, so the width measured at its superficial border (*dashed line*, Figure 2D) was almost always the maximal width. Many ENs had flattened aspect ratios, resembling an oblate spheroid (e.g., Figure 5C), with semi-width > depth in 64% of ENs. In the remainder, the depth exceeded the semi-width, like a prolate spheroid (e.g., AO #4 in Figure 2H). While some ENs were basal and orthogonal to their AO’s long axis (Figure 2D), many ENs were tilted, and some ENs covered only one side of an AO’s lumen (e.g., AO #1 in Figure 2H). A few deep full-size AOs showed an EN of only small dimensions.

In parallel views of bleached skin, some AOs had circular profiles, but others had elliptical elongated profiles (Figures 2K,L). We measured as ∼1.35 their mean ellipticity, the ratio of major to minor axes, from ellipses fitted in ImageJ to traced outlines of AOs (Table 3A). This mean ellipticity ratio was then used to apportion algebraically the mean measured width of ENs into calculated major and minor axis ellipse widths, assuming that the mean width tended to be an average of the ellipse axes because cross sections sampled ENs in different orientations.

From these measured means and axis estimates, we modeled the apical EN surface area as two shapes, an oblate hemispheroid (calculated from the measured mean semi-width and depth) or a triaxial hemiellipsoid. The latter’s surface area was calculated from the measured mean depth, along with the estimated major and minor semi-axis widths, using Knud Thomsen’s approximate formula. Results from the two models yielded similar estimates of apical EN surface area (Table 3B).

Dividing by the mean apical EN area per receptor cell (Table 2, #3) yielded similar estimates of 668 -229/+266 or 665 -226/+263 receptor cells per EN for the hemispheroid or hemiellipsoid models, respectively (Table 3B). The SD values were asymmetrical due to the nonlinear squared-like nature of surface area. Large coefficients of variation (34–40%) attested to large variation of EN dimensions.

#### Convergence Ratio

The total number of receptor cells in an average ER on *Polyodon*’s rostrum was estimated as 15,495 ± 6,052 (Table 4B), based on the mean ± SD number of skin openings exciting one afferent, and an estimated mean number of receptor cells per EN (665). This provided a maximal estimate of the morphological convergence ratio of receptor cells onto an individual afferent, assuming 100% innervation (i.e., that an afferent innervated every possible receptor cell), and based on our result from functional mapping that a given afferent innervated every AO within the boundaries of its receptive field. Convergence from smaller subgroups of AOs, rather than the total convergence ratio, may be functionally relevant (see DISCUSSION).

**TABLE 4:** Summary of convergence values.

	(A) Measured: (or modeled)	Value	*v* (from)	(B) Calculated:	Formula	Value
a	# AOs per ER	23.3 ± 9.1, range 6–45	48 afferents	# Receptor cells per ER (max. convergence ratio)	= a ⋅ b	15,495 ± 6,052 (4,122 – 33,000 est.)
b	# Receptor cells per AO	665 (hemiellipsoid)	116 EN	# Receptor cells per heminode	= b ⋅ d	1,742
c	# Afferents per ER	3.08 ± 0.51, range 2–4	12 ERs (4 fish)	# Local groups of AOs per ER	= a / d	∼9
d	# AOs innervated by a final internode	2.62 ± 0.77, range 1–4	13 (6 ERs)			

***(A)** Values were measured directly, or were estimated from a model for the surface area of electrosensory neuroepithelia (Table 3). **(B)** Values were derived from those in (A) according to the formulas listed. AO, ampullary organ. ER, electroreceptor. est., estimated values. max., maximum. #, v, number of values.*

Estimates of total convergence ratio varied widely, given the different types of AOs, the wide range of AO counts in different receptive fields, and the wide range of EN dimensions. Thus, different ERs may have from ∼4,122 receptor cells (for a receptive field with six AOs, each with a single hemi-spheroidal EN of mean dimensions measured from a subsample of 24 small-type AOs), to ∼33,000 receptor cells (for a receptive field with 45 AOs, mean hemiellipsoidal ENs, and 10% large-pore AOs counted as two ENs).

### Overview of Afferent Trees

To analyze how convergence from receptor cells was complemented by divergence of afferents, we imaged the afferent innervation of ERs on *Polyodon*’s rostrum. We report that each afferent’s peripheral terminal at an ER formed three types of arborizations, in serial order at sequential radial distances from the first branch point near the center of a receptive field (Figure 1). From distal to proximal, the branching included: (i) radial unmyelinated arbors on each AO, which connected via parallel fine projection neurites to (ii) inline branching structures in unmyelinated transition zones at multiple extremities of (iii) a central radial myelinated tree. We did not observe interneurons in *Polyodon* ERs.

We imaged afferent trees by immunolabeling sensory neuron processes with neuron-specific anti–neurofilament-H (Nefh) (Table 1); Nefh+ processes were also aTub+. Glial sheaths on myelinated dendrites were revealed by anti–myelin basic protein (MBP). Nodes of Ranvier on afferents and myelinated dendrites were labeled with a “universal” Ab to voltage gated Na_*V*_ ion channels, along with anti–K_*V*_1.1 to common voltage gated K ion channels. We also traced afferent branching by retrograde diffusion of lipophilic DiI from small crystals placed on ENs.

Our descriptions of afferent branching (below) follow the distal-to-proximal flow of electrosensory information, from multiple ENs to each afferent’s central myelinated tree.

### Unmyelinated Local Arbors

#### Radial Arbors on Electrosensory Neuroepithelia

Starting most distally (Figure 1), diverging unmyelinated neurites of parallel afferents formed a radial laminar curviplanar arbor which followed the bowl-like shape of an EN (Figures 5A–D), to innervate receptor cells. Our imaging of radial afferent arbors on ENs was aided by their forming a thin lamina, locally almost flat and two-dimensional (2D). Side views of ENs showed slender afferent processes rising vertically (∼orthogonal to an EN’s width) in a lateral shell (Figures 5B,C). They arose from enlarged trunks on the basolateral surfaces of ENs.

Because our functional mapping showed that a given ALLn afferent was excited by every AO within the borders of its receptive field, presumably the unmyelinated innervation of each AO contained branches of all afferents to an ER. Consistent with this, our images indicated that an EN was innervated by multiple overlapping arbors.

Fine afferent branches on ENs terminated as small bouton-like endings (arrowheads, Figure 5E and also Figures 5C,D), presumably onto receptor cells (Jørgensen et al., 1972). Bouton endings were visible along the superficial borders of ENs (Figure 5E). These boutons were comparable to the postsynaptic endings of vestibular afferents onto type II hair cells (Fernández et al., 1995; Huwe et al., 2015).

**FIGURE 6:**
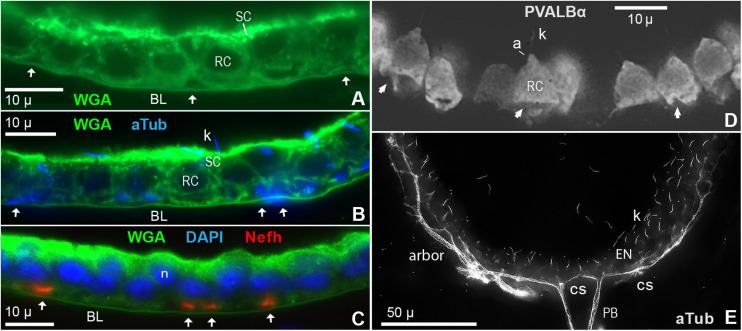
Innervation of electrosensory neuroepithelia (EN). **(A–C)** Cross sections (12 μ) of EN showed unmyelinated afferent arbors occupying triangular basal spaces (arrows) distal to the basal lamina labeled by wheat germ agglutinin (WGA). See keys for labels. RC, Receptor cell. SC, Support cell. **(A)** Basal spaces are marked by arrows; 100x 1.25 NA lens. **(B)** Co-labeling showed that cross-cut afferents labeled by anti–aTub (blue) occupied the basal spaces (arrows); 100x 1.25 NA lens. Anti–aTub also labeled kinocilia (k) on receptor cells. **(C)** Co-labeling showed that cross-cut afferents labeled by anti–Nefh (orange) occupied the basal spaces (arrows); 40x 0.7 NA lens. Nuclei (n) of receptor cells (lower) and support cells (upper) were stained by DAPI. **(D)** Instead of being rounded as expected, the basal poles of many receptor cells (RC), labeled by anti–parvalbumin-α (Table 1), were indented, furrowed, or distorted (arrows), likely by passing trunks of afferent neurites; 60x 1.4 NA lens. a, Apical pyramid on RC. k, Kinocilium on RC. **(E)** Fascicles of projection fibers (PB) approached the basal face of an EN at contact sites (cs), then ramified laterally (arbor); 60x 1.4 NA lens, 0.25 μ stack step size. The calculated optical lateral resolution for this image of anti–aTub blue fluorescence was ∼0.2 μ (Rayleigh criterion) to ∼0.3 μ (for three camera pixels).

In some cross sections of ENs, fascicles of neurites colocalized with basal triangular spaces in ENs (arrows, Figures 6A–C) which were basal to receptor cells, and in-between the basal pedicels of support cells. For example, Figure 6C shows basal spaces containing fascicles of Nefh+ neurites. The neurites were distal to the basal lamina (*BL*, Figures 6A–C) that delimits each EN (Jørgensen et al., 1972), labeled by wheat germ agglutinin lectin (WGA+), consistent with innervation of receptor cells. The furrowed or indented shapes of the basal poles of many receptor cells (arrows, Figure 6D), instead of expected rounded bulbous shapes, were attributed to passing bundles of afferent neurites.

The divergence of neurites in ENs, especially distally, appeared as if it were cellular branching of sensory neuron dendrites, forming conventional unmyelinated postsynaptic dendritic trees (Figure 5). Branches imaged with a 40x 0.7 NA lens were smooth, not obviously varicose. Branches became narrower over sequential branch points. The afferent “branching” on ENs was typically binary, although non-binary branching (i.e., with ≥3 progeny) was observed from enlarged basolateral trunks. Some fine Nefh+ processes were barely resolved by a 60x 1.4 NA lens.

We characterized the afferent “branch points” in ENs by measuring the widths of parent (*D*) and progeny (*d*) branches for a sample of symmetrical branch points of medium-size dendrites (Table 2, #5, #6), not including fine terminal processes or enlarged basolateral processes. The mean width ratio of progeny branches was *d/D* = 0.74 ± 0.10 (*v* = 72), each normalized to its parent, at the high end of the range observed for other vertebrate dendritic branching (Wang et al., 2016). An alternate interpretation suggested that the “branching” in ENs might instead represent defasciculation of fine neurites (see DISCUSSION).

We also consistently observed broad arrays of circular profiles having scalloped edges in *en face* images of ENs (*s*, Figure 5F), by autofluorescence at blue, green, and red wavelengths. We had examples where the cryostat blade dislodged some receptor cells, and in their places were such autofluorescent socket-like profiles. They may represent special interstitial matrix that binds each receptor cell in place.

#### Enlarged Basolateral Segments

On the basal face of ENs, we observed enlarged unmyelinated linear profiles, Nefh+ and aTub+, which extended laterally (Figures 5G–I). A key feature of the enlarged basolateral segments was that each led into several finer Nefh+ “branches” both proximally and distally. That is, projection fibers led into them proximally, whereas distally the basolateral trunks gave rise to slender vertical dendrites on the lateral surfaces of ENs (as Figures 5B–D). For example, Figure 5G shows a discrete segment (*), of 2.1–2.7-μ width and 22-μ length, at the base of several highly branched arbors distally (*red arrows*) which presumably conducted excitatory postsynaptic potentials (EPSPs) from receptor cells. This enlarged segment (*) was at the distal end of three finer projection fibers (*white arrows*), identified from the complete image. The cross-sectional area (4.4 μ^2^) of the enlarged segment was 2.1x larger than the summed cross-sectional area of its projection fibers (calculated from their widths).

Basolateral trunks may correspond to the neurite fascicles observed in some cross sections (Figures 6A–C). Higher resolution images (Figures 5F, 6E, 8C) suggested that basolateral trunks contained parallel neurites, consistent with the trunks being fascicles. Possible models for the basolateral trunks are considered in the DISCUSSION.

#### Basal Contact Sites

**FIGURE 7:**
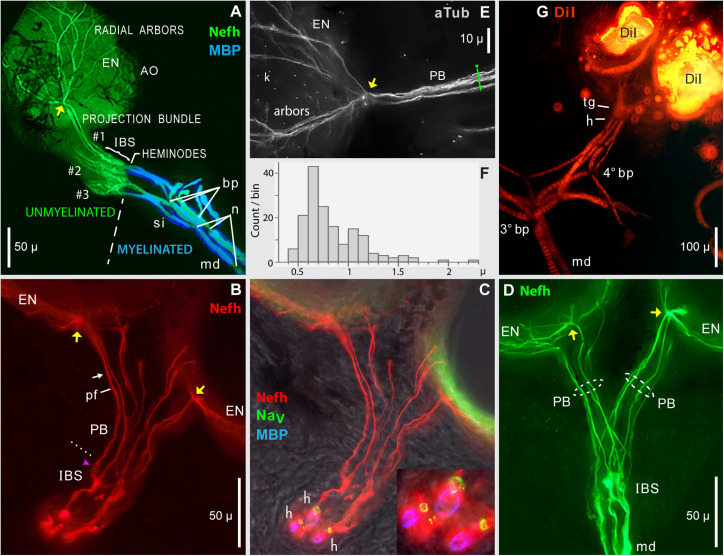
Projection bundles, and transitions to myelinated arbors. **(A–E)** Frozen sections (50 μ) of skin from the rostrum base were immunolabeled to reveal the innervation of ampullary organs by ALLn sensory neuron processes labeled by anti–neurofilament-H (Nefh) or anti–acetylated α-tubulin (aTub). Sheaths on afferent dendrites were revealed by anti–myelin basic protein (MBP). Heminodes (h) were labeled by anti–pan Na_*V*_ (blue), or corresponded to the distal-most edge of MBP+ labeling. yellow arrows, Focal contact sites of projection bundle fibers, on the basolateral face of AOs. **(A)** Overview of the afferent innervation of ampullary organs (AO). The dashed line marks the transition from myelinated dendrites (md, blue) to unmyelinated arbors (green), at inline branching structures (IBS) where afferents divided into parallel projection bundles (#1, #2, #3) of submicron unmyelinated neurites, which diverged into radial arbors shown partially on one AO, to innervate receptor cells in the electrosensory neuroepithelium (EN). Projection bundles #2 and #3 were truncated; the AOs they innervated were outside the section. bp, Branch points. n, Narrowed nodes of Ranvier. si, Short internodes. **(B,C)** Projection bundles (PB) connected IBSs to ENs. **(B)** dotted line, Transition from IBS to projection fibers (pf). 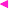, Globular IBS substructure which generated one projection fiber. arrow, Site of Figure 8A. **(C)** Heminodes (h) were labeled (inset) as rings of anti–pan Na_*V*_ labeling (green) encircling three of the afferents, confirmed by the start of MBP labeling (blue) on myelinated dendrites (md). **(D)** Separate projection bundles (PB, dashed ovals) connected one IBS complex to two ampullary organs (AO). md, Large-diameter myelinated dendrites. **(E)** In this enlarged image of a contact site (yellow arrow), fibers in a projection bundle approached an AO, then radiated on its electrosensory neuroepithelium (arbor, EN); blue anti–aTub fluorescence. Kinocilia (k) of receptor cells were also labeled by anti–aTub. **(F)** Histogram of widths of 162 different projection fibers, from 20 images including **(A–E)**, each resampled 4x. **(G)** DiI tracing of distal afferent branches by transcellular retrograde diffusion of lipophilic DiI, from small crystals placed on the EN of two adjacent ampullary organs. Migrating proximally, DiI tracing revealed candidate terminal glia (tg), sites where myelination began at presumed heminodes (h), and third- or fourth-generation branch points (3° bp, 4° bp) of myelinated dendrites (md), which appeared banded due to external melanin (Russell et al., 2019). The 4° branch point had three progeny branches. **(A,G)** 10x 0.3 NA lens. **(B–D)** 40x 0.7 NA lens. **(E)** 40X 0.75 NA water lens.

On the basal face of ENs were “contact sites” (*cs*, Figures 5A–C; yellow arrows, Figures 7A–E) where radial afferent arbors converged, and where “projection fibers” or fascicles thereof departed and connected to the myelinated arbors proximally. Contact sites could be adjacent and centric (Figure 5D), but in many ENs the contact sites were dispersed over the basal face (Figures 5B,C).

Near contact sites, projection fibers must penetrate the substantial melanin-pigmented connective tissue sheath surrounding each AO (Figures 2B,E, 4A, 5A). Afferent transit through the sheath corresponded to short condensed fascicles of projection fibers orthogonal to the basal surface of ENs (Figures 6E, 7E), and basal surface marks on some ENs (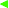, Figure 5D).

#### Projection Fibers and Bundles

**FIGURE 8:**
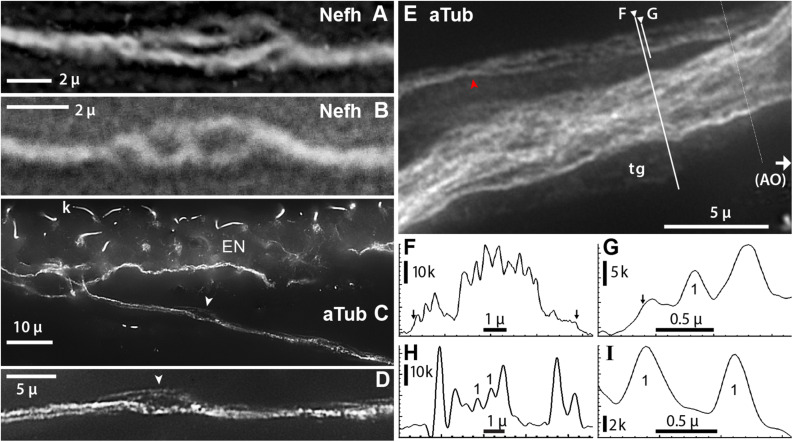
Projection fibers and neurites were labeled by anti–acetylated α-tubulin (aTub, blue emission) or anti–neurofilament-H (Nefh). **(A,B)** Inline sites on projection fibers where neurites separated. **(A)** Enlarged image of a site along one projection fiber, corresponding to arrow in Figure 7B, which locally gave rise to four to six smaller-diameter neurites; 40x 0.7 NA lens. **(B)** This site along a projection fiber exposed two smaller-diameter strands; 40x 0.7 NA lens. **(C,D)** A fascicle near an electrosensory neuroepithelium (EN), separated locally to reveal unitary neurites (arrowhead, **D**); 60x 1.4 NA lens, 0.25 μ steps. Kinocilia (k) on receptor cells were also labeled by anti–aTub. **(E)** A condensed fascicle near an ampullary organ (AO) was accompanied by an individual projection fiber (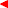) which separated into parallel neurites; 100x 1.25 NA lens, 0.25 μ steps. A terminal glia (tg) sheath surrounded these fascicles; its edges correspond to arrows in (F,G). **(F,G)** Profile plots of fluorescence intensity along lines across projection fascicles, identified on **(E)**; plots started at ◀. **(G–I)** Profile plots showed small peaks attributed to unitary neurites (“1”). **(H)** Profile plot along the green line in Figure 7E; 40x 0.75 NA water lens. **(I)** Profile plot across two small neurites in another image; 60x 1.4 NA lens. The widths of these two peaks, measured near their base at 15% of peak height above extrapolated baseline, were 0.41, 0.35 μ. **(C–I)** From 16-bit monochrome images of blue fluorescence. k, x1000 pixel level.

A single “projection bundle” (*PB*, Figure 7) of afferent projection fibers connected the basal face of each EN to the myelinated arbors proximally. The projection fibers within such bundles were imaged as long fine Nefh+ or aTub+ linear profiles, loosely packed together and approximately parallel (Table 2, #7). Individual projection fibers had total lengths of 105 ± 19 μ (range 64–152 μ), measured segment-wise (*v* = 60 from 16 ERs). The distribution of fiber widths (Figure 7F and Table 2, #8) showed modal widths of 0.6–0.7 μ. Large “widths” were due to visible pairs or fascicles of fibers. Projection bundles followed approximately direct paths to AOs (Figure 7), but often the projection bundles to adjacent AOs could form a “Y” shape (Figure 7B), diverging at midlength but not branching there. Near an AO, the fibers in projection bundles reorganized and tended to condense into fascicles (Figures 6E, 7E, 8E).

The number of projection fibers per projection bundle, which is the average number contacting one EN, was difficult to count because fibers could separate, recombine, pair, or condense *en route* to an AO. Attempts yielded counts of 6.1 ± 1.3 (range 5–9, *v* = 17 from 11 ERs).

Projection fibers were from sensory neurons, as they were labeled by anti–Nefh, a neuron-specific marker, and were traceable to ALLn afferents. The fibers in projection bundles were unmyelinated as they lacked MBP immunoreactivity, and lacked narrowed node-like sites. From many attempts, projection fibers were not labeled by anti–pan Na_*V*_ nor by anti–K_*V*_1.1, consistent with passive membrane properties. While these are negative results, both of these Abs did label ion channels of the myelinated arbors, and nodes on stripped ALLn axons, as positive controls. The pan anti–Na_*V*_ Ab that we used is well-known for labeling a “universal” partial sequence of voltage gated Na ion channels (Gordon et al., 1988; Rasband et al., 1999).

#### Unitary Projection Neurites

Each projection fiber was composed of smaller neurites, <10 in number, based on images of focal sites where a fiber separated into smaller parallel Nefh+ or aTub+ neurites (Figures 8A,B,D). Such inline sites were a few microns long, and occurred anywhere along projection fibers. For example, the fiber in Figure 8A separated locally into four to six neurites. That in Figure 8B separated into two strands. Such images were interpreted as local separation of parallel neurites, not as inline branching.

The widths of unitary projection neurites were 0.37 ± 0.06 μ (Table 2, #9), measured from minor peaks in profile plots across sites where neurites dispersed, including focal inline sites (above), or near AOs (Figures 7E, 8E). Such width values approached the limits of optical microscopy. The peaks (“1” in Figures 8G–I) appeared unitary because they had consistent minimal width and height. The larger peaks in profile plots across projection fascicles (Figures 8F–H) presumably recorded the summed fluorescence of overlapping projection fibers and neurites.

### Transition Zones

The projection fibers and neurites originated proximally in complex transition zones, in-between the unmyelinated and afferent arbors, which included unmyelinated “inline branching structures” (IBSs). Such transition zones, at every extremity of the myelinated arbors, occupied ∼53 μ (Table 2, #10) between the proximal ends of projection fibers and the distal ends of terminal internodes of myelinated dendrites.

#### Inline Branching Structures

**FIGURE 9:**
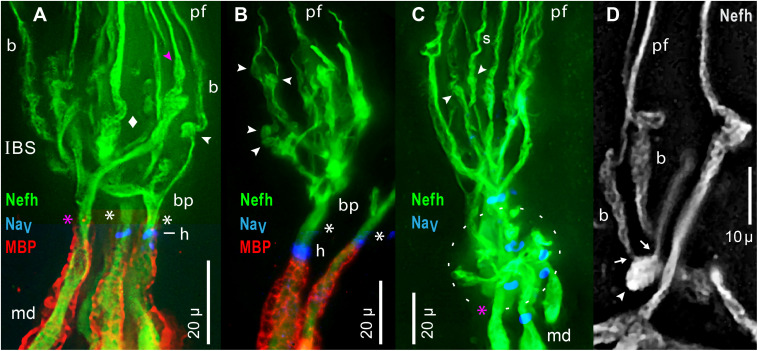
Inline branching structures (IBS), in clusters, were immunolabeled per keys by anti–neurofilament-H (Nefh), or anti–myelin basic protein (MBP), or by a pan Ab to voltage gated Na ion channels (Na_*V*_). **(A)** IBSs included proximal branch points (bp) and thick irregular twisted processes (diamond). **(A–D)** IBSs contained numerous globular ovoid swellings (arrowheads) which gave rise to submicron unmyelinated neurites and projection fibers (pf). In-between them could be segments having a braided (b in **A,D**) or spiral (s in **C**) appearance. Some ovoids tapered distally (pink 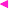 in A), and directly gave rise to a projection fiber, without an intervening braided structure. **(A–C)** A single root afferent process (*), undivided and unmyelinated (MBP–), connected each IBS to the start of a myelinated (MBP+) internode proximally. Some roots (white asterisks) had a Na_*V*_+ heminode (h), but adjacent roots in similar focal planes lacked Na_*V*_ immunoreactivity (pink asterisks in A,C). **(A,B)** Superimposed images of MBP+ and Na_*V*_+ labeling were cropped to include the relevant internode and heminode regions, but not diffuse MBP+ or Na_*V*_+ labeling in overlying terminal glia which obscured the Nefh+ processes in IBS clusters. **(C)** The oval dashed line enclosed an adjacent IBS cluster, viewed end-on, with short lateral dendrites. **(D)** This partial IBS showed Nefh+ afferent neurite bundles (arrows) erupting from an ovoid structure (arrowhead), leading to braided (b) segments, then to projection fibers (pf) of different calibers. Red fluorescence, 4x resampled; the surface mottling of neuronal processes was also visible in single raw images. **(A–D)** 40x 0.7 NA lens.

Each IBS (Figures 9A–D) carried out “inline” branching because its distal branches did not diverge, but instead formed the parallel fibers of projection bundles. A size transition occurred at IBSs, in the diameters of sensory neuron processes, from the “large” (>3 μ) widths of myelinated dendrite neurites, to the submicron diameters of projection neurites distally. The afferent processes in an IBS were unmyelinated (MBP-), but IBSs were enveloped by satellite glial cells (see below). The IBSs occurred in dense clusters (Figures 9A,C) which were complex and varied.

#### Special Components of IBS

The components of an IBS were better-resolved in some examples with fewer parts. Figure 9B shows an individual IBS leading from a separate myelinated dendrite, and Figure 9D shows a partial IBS. Unusual component structures within IBSs included globular structures (arrowheads, Figure 9), of mean dimensions 6.1 × 3.8 μ (Table 2, #11, #12). Such ovoids occurred at middle to distal levels of an IBS (Figure 9), not at the most proximal levels. Our images established that the ovoids were the origin of the long submicron projection neurites and fibers, because close-up views showed that fine fascicles of projection neurites erupted from ovoids (arrows, Figure 9D). Some ovoids were tapered and narrowed at their distal end, from which a projection fiber departed distally (pink 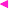, Figures 7B, 9A).

In other examples, *de novo* neurites from ovoids formed braided structures (*b*, Figures 9A,D), ∼15 μ long, having a woven appearance, which could expand distally (Figure 9D), or could spiral (*s*, Figure 9C). A braided structure ended distally in a projection fiber. Neurites from adjacent braided structures could entwine and recombine, sometimes forming a thicker projection fiber (*pf*, Figure 9D) which likely contained extra neurites. The functional role of braided structures was unclear because some projection fibers departed directly from a tapered ovoid structure without an intervening braided structure (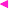, Figures 7B, 9A).

#### Dendrites in IBS

Proximally in each IBS were unmyelinated branch points (*bp*, Figures 9A,B). Some branches were thick trunks with irregular swellings (diamond, Figure 9A), which were twisted and had a plaited appearance (Figures 9A,D), including in single raw images. Swellings on trunks preceded the ovoids described above. Hence, within an IBS, there was often a sequence of (proximal) branch points, trunks, swellings, ovoids, braided structures, and then projection fibers distally.

#### Convergence From Local Groups of AOs

The one to four projection bundles which continued distally from each IBS cluster (transition zone) co-innervated a “local group” of 2.62 ± 0.77 adjacent AOs (range 1–4) (Figures 7B,D,G and Table 4A, line d). To obtain these values, we traced complete unmyelinated projections from transition zones to AOs, in parallel images of skin wholemounts. Discrete local groups of AOs formed the perimeter of rosettes (*LG*, Figure 2L) or could be grouped into higher-order subclusters (Figure 2K). Afferent innervation often followed the spaces between AO groups.

#### Roots of Unmyelinated Arbors

An important component of each IBS was an enlarged sensory neuron process (asterisks, Figures 9A–C), undivided and unmyelinated, which led proximally. Its significance was that it was the output site of an IBS’s unmyelinated arbor, and likely conducted summed EPSPs from ∼1,742 receptor cells of one local group of AOs (Table 4B, line b; assuming 100% innervation).

The mean path length of root segments was 10.3 μ, from the most proximal branch point of an IBS to the start of myelin (Table 2, #13). Their mean width near the start of myelin was 4.3 μ (Table 2, #14). Such root segments usually tapered to a narrowest point near midlength (Table 2, #15, #16), resembling the axon initial segments of CNS neurons (Nelson and Jenkins, 2017).

#### Candidate Spike Generation Sites

Each IBS root segment led into a MBP+ dendrite, at an extremity of the central myelinated afferent trees. Near the start of MBP+ labeling (Figures 9A,B), many (but not all) of the unmyelinated root segments expressed a focal ring of pan Na_*V*_ positive immunoreactivity (i.e., voltage gated Na ion channels), and so formed a heminode (*h*, Figures 9A–C; inset, Figure 7C). Such candidate spike generation sites resembled the heminodes of afferents to other sensory receptors (e.g., Carrasco et al., 2017). However, some roots of unmyelinated arbors appeared to not express pan Na_*V*_ immunoreactivity (pink asterisks, Figures 9A,C).

We also observed an alternate structural candidate for spike initiation sites, a “branch point” type, with no or few heminodes on the roots of IBSs. Instead, pan Na_*V*_ positive immunoreactivity increased in density at nodal sites along a series of 5°, 4°, and 3° distal MBP+ dendrites, in a local arbor at an extremity of myelinated trees. Further data on spike initiation zones (SIZs) would exceed the scope of this report.

### Terminal Glia

**FIGURE 10:**
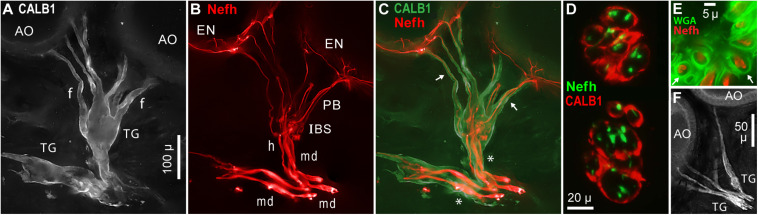
Terminal glia encased unmyelinated arbors. **(A)** Two terminal glial (TG) cells were labeled by anti–calbindin (CALB1); monochrome fluorescence. These terminal glia projected finger-like processes (f) toward ampullary organs (AO). **(B)** Parallel afferents were labeled by anti–neurofilament-H (Nefh), pseudocolored red, including short segments of enlarged afferent myelinated dendrites (md), a tapered zone of inferred heminodes (h), inline branching structures (IBS), unmyelinated fibers of projection bundles (PB), and radial arbors in electrosensory neuroepithelia (EN). **(C)** Merged view of **(A,B)**, pseudocolored. Calbindin+ glial fingers (arrows) visibly enclosed Nefh+ neurites. *, Glia-on-glia organization; see RESULTS text. **(D)** Sections across two IBSs showed CALB1+ terminal glial folds surrounding Nefh+ sensory neuron processes. **(E)** Section across a projection bundle, pseudocolored. The outer sheaths (basal lamina, arrows) of terminal glia fingers were labeled by wheat germ agglutinin (WGA). Subdivisions of the Nefh+ afferent fibers are visible. **(F)** Anti–NMDAR1 labeling of terminal glial (TG). **(A–C)** 20x 0.5 NA lens. **(D)** 40x 0.7 NA lens. **(E)** 100x 1.25 NA lens. **(F)** 10x 0.3 NA lens.

Large terminal (satellite) glial cells were associated with the unmyelinated transition zones and projection fibers of afferents to *Polyodon* AOs. An effective label for terminal glia was a polyclonal Ab to a partial sequence of human calbindin (CALB1, Table 1 and Figure 10A), which outlined the cell membrane. Comparison to afferent neurofilament-H labeling (Figure 10B) showed that the bulbous CALB1+ soma of one terminal glial cell enveloped topologically an entire transition zone complex of IBSs (Figure 10C). Sections across IBSs showed CALB1+ glial folds around the Nefh+ sensory neuron profiles within (Figure 10D).

Finger-like extensions of terminal glia formed sheaths around the fibers of projection bundles (*f*, Figures 10A,C). Glial tendrils stopped at, and did not enter, the basal face of an EN. An individual terminal glial cell could project tendrils to more than one adjacent AOs (Figures 10A,C).

The base of a terminal glia cell enclosed part of the final “large”-diameter myelinated dendrites of afferents (*md*, Figure 10B), proximal to heminodes. That is, the myelin Schwann cells on final short afferent internodes were surrounded by a different type of glia, a terminal glial cell, in an example of “glia-on-glia” organization (*, Figure 10C).

A basal lamina on each terminal glia was revealed by WGA or peanut agglutinin lectins. Sections through projection bundles showed WGA+ labeling of an outer basal lamina on glial tendrils (arrows, Figure 10E). Both lectins similarly labeled the basal lamina of myelin sheaths on myelinated dendrites, and on stripped ALLn axons.

Other antibodies known to label terminal glial cells (Woo et al., 2012; Table 1) also labeled the terminal glia of *Polyodon* ERs. Effective probes included (i) anti–NMDAR1 (Figure 10F), a subunit of glutamate receptors binding *N*-methyl-D-aspartate agonist, (ii) anti–S100β, a subunit of another calcium binding protein, and (iii) anti–*N*-cadherin, a protein of common intercellular junctions (Geiger et al., 1990; Kaidoh and Inoué, 2008). Anti–pan Na_*V*_, anti–K_*V*_1.1, and anti–MBP labeled diffusely within the terminal glia, but did not label their cell membranes, or only faintly so. Anti–Protein zero labeled the terminal glia.

### Myelinated Radial Central Arbors

#### Overview of Myelinated Arbors

The local unmyelinated arbors described above were at multiple extremities of the central star-like myelinated arbor of each of a few parallel ALLn afferents. The myelinated arbors at ERs on the rostrum are a complex topic, surveyed here to provide context for our more detailed analysis of unmyelinated arbors. Myelinated arbors spanned an entire ER receptive field, ∼0.5 to ∼2 mm across. Our functional mapping (above) showed that an ER’s myelinated arbor collected electrosensory input from all AOs within the sharp borders of a receptive field. Our imaging (here) showed that myelinated branching began from a first (1°) branch point near the center of a receptive field, at a subcutaneous depth of 600–800 μ. From it, a few large-diameter first-generation (gen1) myelinated branches projected toward subdivisions of a receptive field, and then underwent a few more generations of myelinated branching in radial serial stages. The branches may be termed “myelinated dendrites” because they perform a dendritic function of collecting electrosensory input. With (candidate) spike initiation sites at each distal extremity, the myelinated tree of each afferent had multiple (candidate) spike generators, in a star-topology configuration. Other sensory receptors also have multiple spike generators (Eagles and Purple, 1974; Lesniak et al., 2014).

#### Counts of ALLn Afferents per ER

**FIGURE 11:**
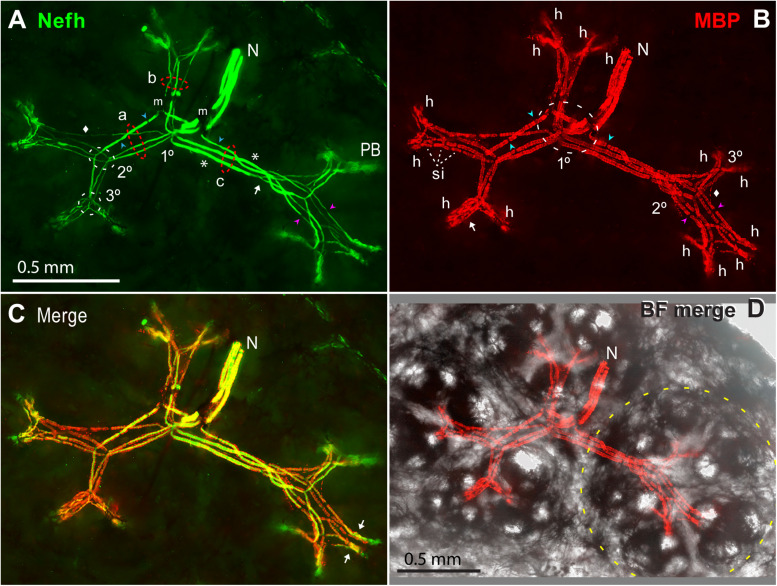
Myelinated afferent arbors of one electroreceptor’s large multipart receptive field on the base of the rostrum. The underside was imaged *en bloc* (4x 0.13 NA lens) in a thick section parallel to skin, as stack projections. **(A)** The small nerve (N) with three ALLn sensory neuron axons (Nefh+) of similar large diameter entered the receptive field near its center. 1, 2, 3°, First, second, or third branch points along myelinated dendrites, proximal-to-distal. Red dashed ovals mark three bundles (a, b, c) of long first-generation myelinated dendrites; each dendritic bundle included three wide and one thin dendrites. These “gen1” dendrites arose from 1° branch points, and projected to different subclusters of ampullary organs. Gen1 dendrites had 0 (*) or 1 or 2 (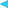) nodes of Ranvier, and ended at 2° branch points (2° white dashed oval). Myelinated branch points were networks of narrowed nodes of Ranvier (e.g., arrow near bundle c). The 2° dendrites had nodes (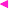), and diverged to nearby 3° branch points (3° white dashed oval). m, Melanin obscured some sites. PB, Projection bundles. **(B)** Myelin sheaths on afferent dendrite internodes were labeled by anti–myelin basic protein (MBP). Fluorescent background was removed for clarity. The white dashed oval (1°) enclosed a zone of first branch points. Gaps in myelin (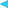, 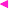) corresponded to nodes of Ranvier marked in **(A)**. Arrow, Parallel MBP+ dendrites of a local gen3,4,5 arbor. h, All 14 candidate spike initiation zones are marked, where MBP+ labeling stopped. si, Short internodes on 2° dendrites. **(A,B)** Diamonds mark thin MBP+ branches. **(C)** Superimposed Nefh+ and MBP+ labeling. Arrows mark single gen3,4,5 arbors, not V-shaped. **(D)** Correspondence of afferent arbors to the receptive field. MBP+ afferent labeling (as **B**) was superimposed on a transilluminated brightfield (BF) image of four rosettes of ampullary organs, one outlined by the yellow dashed line. Small bright circles were skin pores of ampullary organs.

Our imaging showed that a small ALLn branch nerve (e.g., *N*, Figure 11) with 3.08 ± 0.51 parallel ALLn afferents (range 2–4; Table 4A, line c) entered an ER’s receptive field near its center. It derived from ALLn branches in deeper tissue of the rostrum interior (Allis, 1920; Norris, 1925). We observed other nerves traveling parallel to the rostrum’s skin, slightly below it, but they did not contain ER afferents.

The afferent fibers in small ALLn nerves near ERs had large and uniform areas in cross sections. Their Nefh+ sensory neuron axons had equivalent diameters (for a circle of equal area) of 14.8 ± 1.6 μ (*v* = 39, from four fish of ∼1-year age). These had myelin sheaths of 21.5 ± 1.6 μ outer equivalent diameters. Older/longer fish had larger afferents. The coefficient of variation of (equivalent) axon diameters in a given nerve was small (6.7–10.1%, for five nerves), as evidence for homogeneous spike conduction velocities in the parallel ALLn afferents to a given ER. Most afferent nerves also contained a few small Nefh+ profiles.

#### Properties of Myelinated Dendrites

The myelinated branches of parallel afferents overlapped and entwined in a lamina that was parallel to skin, ∼200 μ thick at ∼550 to ∼750 μ subsurface depth, below the basal poles of AOs. For example, Figure 11A shows the myelinated afferent arbors for a large multipart receptive field on the rostrum base, innervated by three large afferents. We immunolabeled 16 complete myelinated arbors and many partial arbors in thick sections, with commercial 1° Abs to key marker proteins of peripheral myelinated axons (Table 1).

The myelinated arbors of the ∼3 large parallel afferents to an ER had similar but usually not identical branching patterns. The radiating dendrites of different afferents were parallel in loose bundles (*a*, *b*, *c* in Figure 11A), at every level of myelinated branching. Counts of myelinated branches in proximal dendritic bundles were similar to the number of nerve afferents, but could differ, and counts tended to increase distally.

Although not tapered, progeny branches (from branch points in myelinated arbors) became narrower, to 66 ± 13% of parent width (Table 2, #17), in a stepwise progressive manner over serial branch points, going distally. However, the summed transverse (calculated) area of all progeny branches was usually larger than the respective parental dendrite area (Table 2, #18).

Some branches with MBP+ sheaths were thin (diamonds, Figures 11A,B). They could accompany large-diameter dendrites, or could cross between 3°; arbors or even between the innervation fields of different gen1 bundles. It was unclear if they were supernumerary branches from large afferents, or instead arose from the small-diameter axons in afferent nerves.

##### Myelin sheaths on terminal branches

MBP+ labeling of thick-wall sheaths surrounding the central afferent branches indicated that they were myelinated (Figure 11B). The sheaths’ MBP+ labeling was often plaque-like and discontinuous (Figure 9B) but was continuous in others. We also demonstrated anti–Protein zero labeling of dendrite sheaths. The sheaths had crenulated surfaces (Figure 11B), with a surface lamina revealed by binding of WGA or peanut agglutinin lectins. Dark transverse striations (Figures 7G, 11B) were attributed to melanin (Russell et al., 2019). Proof that dendritic branches were functionally myelinated would require EM demonstration of compact myelin wrappings, as at other afferent terminals (Quick et al., 1979).

##### Nodes on myelinated dendrites

Inline nodes of Ranvier were observed throughout myelinated branches, confirming their spiking nature. Inline nodes corresponded to gaps in myelin sheaths and had focal rings of pan Na_*V*_ labeling flanked by juxtaparanodal shoulders of K_*V*_1.1 labeling extending under myelin, like classical axonal nodes. The inline nodes of large myelinated dendrites were narrowed (arrowheads, Figure 11A), like classical axonal nodes, but nodes were less- or not narrowed on the most distal MBP+ dendrites.

The branch points of myelinated dendrites always corresponded to compound nodes of Ranvier (Quick et al., 1979; Russell et al., 2019), typically with narrowed Nefh+ processes (arrow, Figure 11A). Many of the branch points of myelinated arbors were binary, that is, with two progeny branches. However, non-binary branch points with three or more progeny myelinated neuronal processes were observed routinely (Russell et al., 2019). Their ≥3 progeny branches arose in one step, not as serial bifurcations.

Another useful identifier of dendritic nodes (inline or at branch points) was a local region of reduced Nefh immunoreactivity (Figure 11A) (Russell et al., 2019). This low Nefh labeling presumably was due to reduced phosphorylation of neurofilament-H at nodes (Mata et al., 1992), and Ab preference for phosphorylated neurofilament-H.

#### Branching of Myelinated Dendrites

##### Centric 1° branch points

The 1° branch points of the parallel ALLn afferents innervating an ER were collected together near its center. Some 1° branchings were compound, comprising pairs of adjacent branch points in series (1° white dashed oval, Figure 11B). For a small receptive field, the 1° branch points were typically under an “empty” centric area with few or no AOs whereas most AOs were located along a perimeter (Figure 2L). If a receptive field included two to four rosettes of AOs, the 1° branch points were near the center of the ensemble (Figure 11D).

##### Generations of myelinated dendrites

As myelinated arbors radiated from a 1° branch point, serial branch points generated two to five sequential generations of myelinated dendrites.

###### Gen1

The first-generation (gen1) myelinated dendrites of each afferent, two to four in number, radiated distally from its centric 1° branch point. Gen1 dendrites often had one or two inline nodes (blue 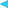, Figure 11A), but some had none (*, Figure 11A). A gen1 dendrite typically ended distally at a 2° branch point (arrow, Figure 11A).

Gen1 dendrites were always the longest and widest afferent dendrites. The dendritic bundles of gen1 branches of parallel afferents (*a*, *b*, *c* in Figure 11A) were directed along straight paths in different planar directions, parallel to skin. For a receptive field with one rosette of AOs, the gen1 dendrites projected to different parts of the rosette. For large multipart ERs, the gen1 dendrites projected to different rosettes (Figure 11D). Thus, bundles of gen1 dendrites created the basic shape of myelinated arbors, projecting to subgroups of AOs.

The branching of gen1 myelinated dendrites included many variants. For example, some elongated ERs had an additional level of branching along gen1 dendrites. Also, a gen1 dendrite could lead to a 3°-type arbor without proximal 2° branching.

###### Gen2

The second-generation (gen2) myelinated dendrites were shorter and thinner than gen1 dendrites, but varied in diameter. They were progeny of 2° branch points, located adjacently for parallel afferents (2° white dashed oval, Figure 11A), near a side of an AO rosette. Each 2° myelinated dendrite usually led to a 3° branch point, but some 2° dendrites ended at a heminode without further branching. 2° myelinated dendrites had inline nodes (pink 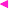, Figure 11A), and often showed multiple serial short internodes with visible gaps (*si*, Figures 7A, 11B).

###### Gen3,4,5 arbors

The third-generation (gen3) myelinated dendrites were progeny of 3° branch points, grouped together for parallel afferents (3° white dashed oval, Figures 7G, 11A). Gen3 dendrites were shorter than gen2 dendrites, and projected toward nearby subgroups of AOs. Gen3 dendrites often led to shorter gen4 and gen5 branches showing modified and thinner MBP+ glial sheaths, in distal local gen3,4,5 arbors (arrow, Figure 11B). Many 3° branch points were binary, and from their two progeny gen3 dendrites led a pair of gen3,4,5 arbors in a V-shape, e.g., with ∼120-μ-long limbs in Figure 11. Other sub-arbors occurred singly (arrows, Figure 11C). The gen3,4,5 arbors, overlapping for parallel afferents, could be complex.

In summary, gen1 myelinated dendrites projected to major parts of a receptive field, then shorter MBP+ gen2 and gen3,4,5 dendrites projected to near subgroups of AOs, from where local unmyelinated arbors projected to local groups of AOs and their receptor cells.

## Discussion

Convergence is a striking feature of electroreceptors on the rostrum of *Polyodon*, that we analyzed by functional mapping of receptive fields, estimating component numbers, and imaging of afferent arbors. The two-stage ERs of *Polyodon* present experimental advantages as a model system for studying sensory convergence because they lack interneurons and associated lateral synaptic circuitry, and the skin pores allow access to transducers. Our report increases the understanding of convergence in sensory receptors. Convergence creates star-topology neural networks, of iterative parallel organization, that may yield special functional properties.

We found that ERs on the rostrum had a laminar stratified organization, with ∼1-mm depth. This differed from the flattened organization of specialized ERs in the thin flexible hypobranchial skin (Kistler, 1906; Wilkens et al., 2002).

### Convergence in *Polyodon* Electroreceptors

Table 4A collects our measured values for convergence in *Polyodon* ERs. From these, other quantities relevant to convergence were calculated (Table 4B). An ER on *Polyodon*’s rostrum includes a unique set of 23.3 ± 9.1 AOs and a unique set of 3.08 ± 0.51 myelinated ALLn afferents, innervating only those AOs, based on our functional mapping and afferent imaging data. Receptive fields of afferents correspond to natural morphological clusters of AOs. Other ERs show similar clustered AO organization (Peters and Mast, 1983).

Our report explicitly quantitates the total number of receptor cells in a Lorenzinian-type electroreceptor, as 15,495 ± 6,052 receptors cells (Table 4B) in the cluster of AOs comprising an afferent’s receptive field on *Polyodon*’s rostrum. This may be the total morphological convergence ratio of receptor cells onto individual afferents, because we showed by functional mapping that an afferent innervates every AO within the borders of its receptive field, although whether an afferent innervates 100% of receptor cells per AO remains unproven. However, functional convergence may differ from total morphological convergence (see below).

Our estimates of the number of receptor cell per EN were based on the mean measured EN width, and hence may be underestimates due to nonlinearity of EN surface area. For example, calculating the hemispheroidal areas of individual ENs, then averaging, yielded an estimate of 708 receptor cells per EN, ∼6% larger than in Table 3.

Reviews on the ampulla of Lorenzini ER of Chondrichthyes, comprising a single large AO, have suggested ∼10,000 receptors cells per ER as a rough estimate (Petracchi and Cercignani, 1998; Kalmijn, 2000). Their repute for high electrosensitivity points to a relation to their large-scale convergence. Receptor cells have been counted in ampullary organs of catfish (e.g., Whitehead et al., 2009).

#### Convergence May Increase SNR

Convergence ratios in several types of sensory receptors support a positive correlation between sensory sensitivity and the number of sensors (Corwin, 1983; Meisami, 1989; Sanchez and Zakon, 1990; Peters et al., 1997). The effects of convergence on signal processing have been studied extensively in retinal cells of vertebrates (Baden et al., 2018), involving several neural mechanisms. (i) Higher convergence is associated with vision in dim light (Rodieck, 1998; Litherland and Collin, 2008; Querubin et al., 2009). (ii) Special problems of single-photon responses (Pahlberg and Sampath, 2011) are not relevant to electrosensory transduction in ampullary ERs because the expanded ampulla and conductive gel of AOs likely conduct an external stimulus uniformly to receptor cells. (iii) Dark noise in photoreceptors is reduced by electrical coupling among neighbors (Lamb and Simon, 1976; Zhang and Wu, 2004). This partial noise cancelation is similar to the “parallel signal averaging” used in analog electronic arrays of parallel sensors (Wikswo et al., 1983; Mohammadi et al., 2015).

The large receptor cell arrays in *Polyodon* ERs and other Lorenzinian ERs may increase the SNR of afferent output, and hence increase their receiver sensitivity (Pettai, 1984; Avionics Department, 2013). The numerous receptor cells will presumably increase the total correlated synaptic EPSP power evoked by a common external stimulus. Noise affecting Lorenzinian ERs (Adair et al., 1998) likely arises at several stages, including source, transduction, and synaptic noises. The uncorrelated internal noises of different receptor cells may partly cancel. Thus, external signals may be averaged in parallel across numerous receptor cells, increasing the SNR of their aggregate output (Petracchi and Cercignani, 1998; Kalmijn, 2000). However, these theoretical proposals remain unproven experimentally.

#### *Polyodon* ERs as Sensory Arrays

Because multiple AOs form a perimeter or fill a planar area in an afferent’s clustered receptive field, each *Polyodon* ER is a 2D sensor array, treating each AO as a “sensor.”

A major advantage of electronic sensor arrays is “beamforming,” whereby phase lags or time differences of arrival to different sensors allow source localization (van Trees, 2002). Small paddlefish strike at individual planktonic prey, so source localization is vital to their particulate mode of foraging. Nevertheless, a *Polyodon* ER is incapable of conventional beamforming directivity because electrical signals propagate at electromagnetic wave velocity, an ER’s array has millimeter scale, and a postsynaptic afferent is a hardwired common output from “sensors” of fixed weightings. Thus, electrical signals from a distant small individual prey (e.g., *Daphnia*) likely stimulate uniformly and simultaneously all AOs in a *Polyodon* ER, depending on the radial distance (Wilkens et al., 2001). Moving near-field prey may stimulate ERs more complexly.

The *m* parallel transducers of electronic sensor arrays increase receiver sensitivity via increased signal transduction power, and partial cancelation of uncorrelated noises in parallel transducers. Similar parallel averaging may occur in neural sensory receptors with converging receptor cells. What value should be taken for *m* in *Polyodon* ERs? A simple model (see below) would group together all 4,100–33,000 receptor cells. This would treat a *Polyodon* ER like an ampulla of Lorenzini ER, which comprises a single large AO, in which parallel averaging may operate over numerous receptor cells (Petracchi and Cercignani, 1998), possibly all.

However, lumping together all receptor cells of a *Polyodon* ER may be inappropriate, because it disregards their multiple AOs, the serial unmyelinated/myelinated organization of afferent terminal branching, and putative sensory processing in myelinated arbors. Taking *m* as the estimated number (∼665) of receptor cells in one average AO would ignore our finding that the root of an unmyelinated arbor connects to a local group of ∼2.62 AOs (Table 4A, line d). Hence, for spike initiation in myelinated dendrites, the relevant *m* for parallel averaging among receptor cells may be the estimated ∼1,742 (Table 4B, line b) receptor cells of a local AO group converging onto a heminode SIZ (assuming 100% innervation), or more for a “branch point” SIZ.

We conclude that an average *Polyodon* ER forms a star-topology sensor array of ∼9 functional subgroups of AOs (Table 4B, line c), each with ∼1,742 or more receptor cells, converging onto myelinated afferent trees. Convergence in an ER likely enhances its local signal detection; linear arrays of ERs along the rostrum length mediate spatial discrimination.

### Three Serial Types of Afferent Branching

We analyzed how afferents to ERs on *Polyodon*’s rostrum branch to mediate large-scale convergence. We identified three distinct types of sensory neuron arborizations, including centric myelinated arbors, inline unmyelinated branching, and radial unmyelinated arbors on each AO. Each type formed spatially delimited ensembles of dendrites and branch points, stratified at different tissue depths, and concatenated serially along every afferent path from a first branch point (deepest) to postsynaptic sites on receptor cells (most superficial). Being in series, the divergence ratio of each stage of branching would multiply. This multiplicative serial branching explains how each afferent generates sufficient divergence to innervate up to ∼33,000 receptor cells.

In ampulla of Lorenzini ERs, Metcalf (1916) and Kantner et al. (1962) described the innervation by staining. Waltman (1966) depicted the sheaths of unbranched myelinated afferents as ending in a central region. Hence, these ERs may lack myelinated arbors, and their extensive terminal arbors may be unmyelinated and have terminal glia, as we found at *Polyodon* AOs.

Concepts of network topology (Wilson, 2010) apply to the afferent trees of *Polyodon* ERs. Thus an ALLn axon approaching its first branch point would be termed the afferent terminal’s “root,” radial branching from this hub forms “star” networks, branch points would be network “nodes,” heminodes would be the terminal “leaves” of myelinated trees, the large unmyelinated process continuing from each heminode would be the “root” of one unmyelinated arbor, and postsynaptic bouton contacts with receptor cells would be the “leaves” of unmyelinated arbors.

#### Unmyelinated Arbors of AOs

Many questions remain about the unmyelinated innervation of receptor cells in *Polyodon* AOs, which includes the radial arbors on EN, the enlarged basal trunks near contact sites, the projection fibers, and the proximal IBSs. Our report presents immunolabeling and imaging of unmyelinated afferent arbors, and their terminal (satellite) glia, at Lorenzinian AOs.

A given AO was innervated by usually only one projection bundle. We never observed an AO with several projection bundles leading to different ERs. This supports our definition of a *Polyodon* ER as including a unique set of AOs.

The conduction of EPSPs from receptor cells may occur passively in unmyelinated afferent arbors, as indicated by their negative immunoreactivity for two common voltage gated ion channels (pan Na_*V*_ and K_*V*_1.1). Projection fibers also lacked other signs of nodes, for example, constrictions with locally reduced anti–Nefh immunoreactivity.

Calculations of passive conduction in the long submicron unmyelinated neurites of another sensory receptor (Carr et al., 2009) support the plausibility of passive conduction of EPSPs in the unmyelinated afferent arbors of *Polyodon* ERs, with neurites of comparable length and diameter. The total path lengths of fine neurites, from receptor cells to IBSs, ranged from ∼70 μ (for the shortest observed projection fibers, and receptor cells located basally in an EN) to ∼200 μ (for mean-length projection fibers, to the superficial EN border in Figure 5C along the traced *red line*), or more in the tallest ENs. The low frequency passband of *Polyodon* ERs (∼8 Hz best frequency) may obviate phase lags along neurite paths of different lengths. If projection neurites are helically wound within fibers, this would increase the path length for hypothesized passive conduction of EPSPs from receptor cells.

The large glial cells encasing the unmyelinated IBSs and projection fibers of *Polyodon* ERs resembled in morphology and peripheral location the terminal glia at mammalian cutaneous hair mechanoreceptors (Kaidoh and Inoué, 2008; Woo et al., 2012). Terminal glia presumably provide support for unmyelinated afferent arbors. The number of terminal glia in an ER may correspond to its count of IBS complexes.

##### Multiple arbors per afferent on ENs

Our imaging suggested numerous parallel unmyelinated radial arbors on each EN. Because we found ∼3 afferents per ER, a simple model might predict ∼3 arbors on each EN. Instead, our imaging indicated that the afferent innervation of ENs is much more complex than this. We counted ∼6 fibers per projection bundle, and observed examples of ∼4 unitary neurites in one fiber (Figure 8A), suggesting that ∼24 neurites (likely an underestimate) innervated a typical EN. If each projection neurite formed an arbor, there would be 24 (or more) afferent arbors per EN, likely overlapping.

##### Branching or unpacking?

Instead of cellular dendritic branching of afferents on ENs, high-resolution images (Figures 5F, 6E, 8C) suggested an alternate interpretation, that afferent “branching” on ENs might represent unpacking (defasciculation) of numerous parallel unitary projection neurites. The neurites would be like those observed in projection fibers, ending at postsynaptic boutons. What appeared as “branch points” on ENs could instead be “separation sites” of parallel subfascicles of projection neurites. If so, then the summed cross-sectional areas of progeny “branches” (*a*_*i*_) should equal the parent’s cross-sectional area (*A*), as Σ*a*_*i*_ /*A* = 1, assuming that unitary neurites keep unchanging widths as they radiate on ENs. From our sample of “branch points” on ENs, the ratio of calculated areas was 1.04 ± 0.25 (*v* = 36), not significantly different from 1. Hence, these data did not exclude a defasciculation model for afferent divergence on ENs.

##### Fusion or fascicles?

We considered alternate models for the enlarged basolateral trunks on ENs as (i) fused segments where projection neurites may merge to form cellular compartments that branch distally into postsynaptic dendrites, or (ii) sites of another level of inline branching of projection neurites, or (iii) collected fascicles of projection neurites from IBSs. If fused and interconnected [model (i)], the basolateral trunks together may form a unified strongly coupled electrotonic compartment, like the large gen1 dendrites of myelinated arbors, as a shared organizational motif.

Examples of “giant” axons arising by neuronal fusion are well-known in invertebrates. Further work is needed to determine whether the enlarged basolateral afferent segments on *Polyodon* EN may be an example of fusion in a vertebrate sensory neuron [model (i) above]. If so, the enlarged segments may reset the progressive proximal-to-distal size reduction of afferent processes at branch points, allowing distal EN dendrites to be wider.

##### Regeneration of unmyelinated arbors?

The unmyelinated arbors may also comprise a system for regeneration and repair of the afferent innervation of ER receptor cells over the course of a paddlefish’s lifespan of up to 40 years. Receptor cells may turn over and be replaced, as in mammalian retinas and olfactory epithelia, or the cutaneous AOs may be damaged by skin infections or abrasions. Hence, repair and remodeling of the afferent innervation of ENs are likely requirements for *Polyodon* ERs. The deeper location of the myelinated afferent arbors, amid subdermal cartilages (*c*, Figure 2B), suggests that myelinated arbors may be more protected and stable.

We showed that globular structures at transition zones were the origin of projection neurites and fibers (Figure 9). These ovoid structures appeared to be specialized neuronal modules which specifically produce fine fascicles of parallel submicron projection neurites, innervating AOs. The ovoids may participate in repair and regeneration of EN innervation. We could not find other reports of comparable permanent neuronal structures. The ovoids may resemble the transitory club endings of regenerating axons of cut nerves (DeFelipe and Jones, 1991).

Neurite fascicles, akin to the projection bundles of *Polyodon* ERs, occur in retinal degeneration models (Jones et al., 2003). The terminal dendrites of mammalian light-touch mechanoreceptors undergo rapid remodeling (Marshall et al., 2016). Sensory reinnervation is a medically important topic for skin grafts and other reconstructive surgery.

#### Transition Zone Ratios

We found that the unmyelinated innervation of AOs originated from dense clusters of overlapping IBSs at transition zones between unmyelinated and myelinated trees. Substantial convergence occurred at transition zones due to inline branching.

Our images of well-separated IBSs, and of a single root segment leading from each IBS to an individual MBP+ internode (Figure 9), indicated that a given IBS and its components and projection fibers and neurites derived from one afferent. Hence, a transition zone cluster contained at least as many IBSs as the count of parallel afferents. Clusters contained more IBSs at gen3,4,5 myelinated arbors due to branching of parallel afferents into more than one terminal internode.

We found a 1:1 correspondence of local AO groups to IBS clusters: each IBS cluster was traceable to one local group of 2.62 ± 0.77 AOs. The estimated ∼1,742 receptor cells in an average local group of AOs (Table 4B, line b; assuming 100% innervation in ENs) converged onto the root of an IBS’s unmyelinated arbor. This provides an estimate of the average number of receptor cells driving the spiking of a heminode SIZ.

The total count of SIZs was of interest as a parameter for models of radial myelinated trees. In some examples, an extremity of a myelinated tree, where MBP+ labeling ceased, showed only a single IBS cluster (e.g., Figure 7D). Assuming 1:1 correspondence of IBS clusters to SIZ, an average myelinated tree had ∼9 extremities and SIZs (Table 4B, line c), from the ratio of the mean AO count (23.3) of receptive fields, to the mean number of AOs (2.62) innervated from a final internode. However, other examples showed more than one IBS clusters at complex extremities of myelinated trees (Figure 7A).

An alternate approach was to equate SIZs to the local arbors of gen3,4,5 myelinated dendrites, because they included final MBP+ internodes. For example, the large myelinated tree in Figure 11B had 14 such sub-arbors (labeled *h*).

#### Radial Myelinated Trees

Our morphological data on the organization of afferent terminals indicated that synaptic excitation from ENs is conducted passively to candidate SIZs at multiple distal extremities of each afferent’s central radial myelinated tree. Hence, spikes likely initiate distally on myelinated dendrites. Other vertebrate sensory receptors also have multiple spike initiation sites due to myelinated terminal branching, as studied in numerous reports (e.g., Eagles and Purple, 1974; Lesniak et al., 2014). Myelinated trees were the only afferent component spanning all AOs in a receptive field.

##### Synchronous spikes in radial myelinated trees

Ottoson and Shepherd (1970) proposed that the radially branched myelinated terminals of afferents at vertebrate muscle spindles undergo synchronous spiking, consistent with the high regularity of background afferent firing (Matthews and Stein, 1969). A model for radially symmetrical star networks proposed that spike initiation at multiple distal SIZs may be coherent due to strong coupling via myelinated dendrites (Kromer et al., 2016, 2017).

The spiking central myelinated arbors of *Polyodon* ER afferents may operate similarly, given their radial organization, wide dendrites, and multiple distal candidate SIZs, forming a star-topology network. Synchronous spikes may initiate distally in myelinated trees. The large-diameter gen1 myelinated dendrites (e.g., ∼10 μ in Figure 11A), and the prominent Fa peak in power spectra of background afferent firing (Figure 3A), are consistent with strong coupling in its star-like myelinated arbor (*One compartment?*, Figure 1).

As an extension to ERs with asymmetrical receptive fields (e.g., with multiple clusters of AOs), not perfectly radial, spike timing may be adjusted by variation of gen1 dendrite diameters. For example, in Figure 11A, the gen1 dendrites in bundle *c*, from the most distant AO rosette, were wider than in bundles *a* and *b*, from closer rosettes. Further analysis would exceed this report’s scope. The short internodes on distal dendrites (*si*, Figures 7A, 11B) may also adjust spike timing in myelinated trees (Ford et al., 2015). Such short internodes have been observed on distal branches of other peripheral (Fried and Erdélyi, 1984) and CNS (Deschênes and Landry, 1980) star-like neuronal arbors.

##### Proposed time-sharing

The myelinated arbor of a *Polyodon* ER afferent may alternate between “digital” and “analog” modes of neurophysiological operation. Our time-sharing proposal is based on the brief duration of an individual synchronous spike, hypothesized to occur throughout an afferent terminal’s myelinated tree. The ionic conductances of one spike would dominate the membrane potential of the tree during only the <5 ms (including after-potentials) spike duration. Thus, the spike duration would occupy <25% of the 20-ms average interspike interval (Figure 3A3) of typical ∼50-Hz background firing.

During the remainder of an interspike interval, e.g., for >15 ms or >75% of average duty cycle, the myelinated tree would presumably be available to sum the EPSP voltages conducted passively from AOs throughout a receptive field. Averaging of summed EPSPs may augment the output SNR. Gaps during spikes may be inconsequential due to likely stimulus insensitivity during afferent spikes.

### Models of Other Converging Sensory Arbors

Reports on other vertebrate sensory receptors with multiple sensors and branched myelinated afferents have proposed competition of multiple spike generators (Fukami, 1980; Banks, 1986; Lesniak et al., 2014), or pacemaker properties of 1° branch points (Querfurth, 2006). The large convergence of some visceral afferents (Spencer et al., 2014) may achieve spatial coverage rather than the increased receiver sensitivity proposed for *Polyodon* ERs. Afferents to hair follicle mechanoreceptors each innervate tens to hundreds of hairs over large receptive fields (Suzuki et al., 2012; Wu et al., 2012; Abraira and Ginty, 2013), but convergence summation models may not apply because separate hairs likely incur different stimuli.

## Materials and Methods

All animal procedures were performed in accordance with the institutional animal care committee’s regulations. Paddlefish purchased commercially were maintained in a large recirculating aquarium system.

### Fluorescent Labeling and Imaging

For antibody or lectin labeling, six paddlefish, 1 to 2+ years old, of indeterminate sex and 25–42 cm eye-to-fork length, were fixed minimally. After anesthesia by intramuscular injection of 0.1 g of alfaxalone/cyclodextrin (CTD Inc.) in saline, a catheter was ligatured into the conus arteriosus for perfusion of the vasculature at high flow rate (>30 mL/min). Exsanguination with isotonic phosphate-buffered saline (PBS) containing heparin anticoagulant and Na nitroprusside vasodilator was followed by perfusion fixation for 15 min with cooled 4% wt/vol formaldehyde (from paraformaldehyde, without methanol), 0.25% wt/vol picric acid, 50 mM NaCl, and 50 mM phosphate buffer, pH 7.5. Soft tissue from the base of the rostrum was then cut into ∼5 × 5 × 5-mm blocks, and immersed in fixative until a total elapsed time of ∼45 min since the start of perfusion fixation, then washed extensively in cold PBS. After cryoprotection in cold 20% wt/vol sucrose, a block was oriented in a mold in a 1:2 mixture of OCT gel and 20% sucrose, snap frozen on dry ice/isopentane without immersion, and stored at −30°C until use. Frozen sections were cut 10–50 μ thick on a motorized cryostat (Reichert-Jung 2800E) using disposable blades (Feather), air dried onto amine-adhesive slides, and stored at −30°C. Thick sections of complete afferent projections, up to 600 μ thick, were cut parallel to skin on a vibratome (Leica VT1000), and kept in saline.

Indirect immunofluorescent labeling using commercial 1° and 2° antibodies (Abs) was conventional. Dialyzed delipidated 5% goat serum was used for blocking, and as carrier for Ab solutions, with 0.1% vol/vol Triton X100 detergent for permeabilization, and antimicrobial Na azide. Final slides were coverslipped in a glycerol-based antifade clearing medium (VectaShield). The 1° Abs used were from chicken, rabbit, or mouse hosts (Table 1). Only a few mouse monoclonal Abs were effective. Each 1° Ab was tested individually before mixing them to label two or three antigens simultaneously, by application to tissue for ∼16 h at 6°C, usually at 5 μg/mL (each) for affinity-purified rabbit polyclonal Abs.

To reduce fluorescent aggregates, we turned to goat 2° Abs from Biotium Inc., stated to be “highly cross-adsorbed” against immunoglobulins of other species. They had conjugated CF fluorochromes emitting maximally at 452, 515, or 614 nm, and were applied to tissue at 1 or 2 μg/mL (each) for ∼90 min at 22°C, or overnight at 6°C for thick vibratome slices. Negative control experiments confirmed the low non-specific binding of 2° Abs when 1° Ab was omitted.

When a red- or green-fluorescent phalloidin conjugate (Biotium or Cytoskeleton Inc.) was included, it was incubated 1:40 along with 2° Abs. For superimposed lectin and Ab labeling, wheat germ agglutinin (Biotium) conjugated to CF488 or CF594 fluor was mixed with 2° Abs and/or phalloidin.

Lipophilic DiI (Thermo, Cat# D282; CAS 41085-99-8) was also used to trace afferent processes by diffusion in membranes. Additional paddlefish (*n* = 4), of indeterminate sex and ∼40-cm eye-to-fork length, were fixed by vascular perfusion with 4% paraformaldehyde pH 7.6 in PBS without picrate or alcohol. Tissue blocks from the rostrum base were stored in fixative. After coating tissue with agarose to restrict contamination, small crystals of DiI were placed manually on electrosensory neuroepithelia of AOs.

The colors shown in illustrations matched the emission fluorescence color, unless noted. A widefield epifluorescence microscope with stepper-motorized focus was a modified Nikon Optiphot-2. It had three single-fluor filter sets (Chroma) with ∼50-nm-wide blue, green, or orange/red emission bands. An electrical shutter blocked the Hg arc except when opened by a software-controlled camera. A Diagnostic Instruments 2048 × 2048 pixel monochrome camera like model IN1410, or a 1600 × 1200 pixel Bayer filter color camera (model IN421), was operated using SPOT software. Both cameras had a Kodak CCD sensor with 7.4 × 7.4 μ pixels. Small alignment offsets between fluorescence color channels were calibrated for each objective lens and filter set using TetraSpeck (Thermo) multifluorescent beads. Images were processed or measured using Adobe Photoshop CS6 or Fiji-ImageJ software. Figures show flattened projections from z-stacks (unless noted) of images at up to 60 tissue depths, from an in-focus search-and-merge algorithm. For most illustrations, images were deblurred by 2D deconvolution, contrast was increased, and gamma = 1. Widefield fluorescence microscopy of tissue sections, as here, with software deconvolution, can achieve superior imaging (Shaw, 2006).

### Functional Mapping

The receptive field of 48 single units, recorded from sensory neuron somata in a left-side ALLn ganglion, were mapped by *in vivo* neurophysiological testing of the electrosensitivity of individual AO pores, in six additional paddlefish of ∼1-y age, indeterminate sex, 33.3 ± 1.8 cm eye-to-fork length, and 17.1 ± 0.4 cm rostrum length (tip to nares). A paddlefish was anesthetized by continuous intramuscular infusion of alfaxalone/cyclodextrin from a syringe pump, while partly immersed upright in a plastic chamber, with a flow of oxygenated water into its mouth for respiration. Water (300 L) from the colony tank, at similar temperature (15–16°C), was recirculated with filtration and cooling.

The cranium was opened to expose the left-side ALLn ganglion. Into it was advanced an aluminasilicate micropipet, filled with 3 M NaCl, to record extracellularly an individual sensory neuron’s continuous background firing, or stimulus modulation thereof.

The electrode for stimulation of an individual pore was an Ag wire comparable in size to ER pores: 76 μ Ag diameter with 114 μ OD Teflon coating (A-M Systems). The tips of two wires were cut square, coated with AgCl in bleach, and shaped into a 2.0 mm vertical dipole. A 71 nF parallel film capacitor reduced noise. The lower wire tip was lowered 100 μ into a pore using a hydraulic micromanipulator. At smaller pores, the wire end’s Teflon shell was pressed lightly onto surrounding skin. The dipole wires led to a linear bipolar stimulus isolator (A-M Systems), driven by 5-Hz sine waves from a function generator. Constant-current stimulus amplitudes were ±1 to ±2.5 nA peak-peak. Differential recording across this dipole (in chamber water) showed ± 125-μV peak-peak sine waves for ±2.5 nA (maximal) stimulation. However, the sinusoidal voltage excursion inside a stimulated AO was unknown and was likely smaller due to shunting via the skin pore.

Receptive fields were mapped on only the dorsal left-side quadrant of the rostrum surface. A recorded unit’s receptive field was located using a mobile small dipole electrode, and 5-Hz sine wave stimulation, seeking audible modulation of afferent firing. Then, a unit could usually be held long enough (∼2 h) to measure the SNR at 5 Hz of every skin opening in a sensitive receptive field cluster, and at selected insensitive pores of all adjacent clusters.

Recorded single-unit afferent firing was digitized using a Cambridge Electronic Devices 16-bit interface and Spike v7 software. Power spectra of afferent firing were computed offline from spike times using Spike v7 programs (LEGEND, Figure 3; Neiman and Russell, 2004). The SNR at 5 Hz was calculated from 1-min data, ∼3,000 spikes, for each stimulated pore (LEGEND, Figure 3).

## Conclusion

We quantitated the large convergence ratio of receptor cells onto afferents innervating the ancestral Lorenzinian electroreceptors of *Polyodon*. Fluorescent imaging revealed that afferent terminals were radial in form, and included three distinct stages of branching. Receptor cells were innervated by complex unmyelinated arbors, which may also carry out regeneration and reinnervation. Central star-topology myelinated trees spanned entire receptive fields. The sensor array and large convergence ratio of *Polyodon* electroreceptors likely increase their receiver sensitivity.

## Data Availability Statement

Datasets generated for this study are included in the article.

## Ethics Statement

The animal study was reviewed and approved by the Institutional Animal Care and Use Committee, Ohio University.

## Author Contributions

DFR designed this research and wrote the manuscript. LN, DFR, WZ, DER, and TW carried out the research. DFR, TW, and LN analyzed the data. All the authors contributed to the article and approved the submitted version.

## Conflict of Interest

The authors declare that the research was conducted in the absence of any commercial or financial relationships that could be construed as a potential conflict of interest.
